# Chromosome-level and haplotype-resolved genome provides insight into the tetraploid hybrid origin of patchouli

**DOI:** 10.1038/s41467-022-31121-w

**Published:** 2022-06-18

**Authors:** Yanting Shen, Wanying Li, Ying Zeng, Zhipeng Li, Yiqiong Chen, Jixiang Zhang, Hong Zhao, Lingfang Feng, Dongming Ma, Xiaolu Mo, Puyue Ouyang, Lili Huang, Zheng Wang, Yuannian Jiao, Hong-bin Wang

**Affiliations:** 1 Institute of Medicinal Plant Physiology and Ecology, School of Pharmaceutical Sciences, Guangzhou University of Chinese Medicine, Guangzhou, China; 2 State Key Laboratory of Plant Cell and Chromosome Engineering, Institute of Genetics and Developmental Biology, Innovative Academy for Seed Design, Chinese Academy of Sciences, Beijing, China; 3 State Key Laboratory of Molecular Developmental Biology, Institute of Genetics and Developmental Biology, Chinese Academy of Sciences, Beijing, China; 4 Key Laboratory of Biology and Genetic Improvement of Horticultural Crops (North China), Ministry of Agriculture, Beijing Key Laboratory of Vegetable Germplasm Improvement, Beijing Vegetable Research Center, Beijing Academy of Agriculture and Forestry Sciences, Beijing, China; 5 Key Laboratory of Chinese Medicinal Resource from Lingnan (Guangzhou University of Chinese Medicine), Ministry of Education, Guangzhou, China; 6 School of Traditional Chinese Medicine, Guangdong Food and Drug Vocational College, Guangzhou, China; 7 State Key Laboratory of Systematic and Evolutionary Botany, Institute of Botany, Chinese Academy of Sciences, Beijing, 100093 China; 8 University of Chinese Academy of Sciences, Beijing, 100049 China; 9 State Key Laboratory of Dampness Syndrome of Chinese Medicine, Guangzhou University of Chinese Medicine, Guangzhou, China

**Keywords:** Conservation genomics, Genome evolution, Plant evolution, Polyploidy in plants

## Abstract

Patchouli (*Pogostemon cablin* (Blanco) Benth.), a member of the Lamiaceae family, is an important aromatic plant that has been widely used in medicine and perfumery. Here, we report a 1.94 Gb chromosome-scale assembly of the patchouli genome (contig N50 = 7.97 Mb). The gene annotation reveals that tandem duplication of sesquiterpene biosynthetic genes may be a major contributor to the biosynthesis of patchouli bioactivity components. We further phase the genome into two distinct subgenomes (A and B), and identify a chromosome substitution event that have occurred between them. Further investigations show that a burst of universal LTR-RTs in the A subgenome lead to the divergence between two subgenomes. However, no significant subgenome dominance is detected. Finally, we track the evolutionary scenario of patchouli including whole genome tetraploidization, subgenome divergency, hybridization, and chromosome substitution, which are the key forces to determine the complexity of patchouli genome. Our work sheds light on the evolutionary history of patchouli and offers unprecedented genomic resources for fundamental patchouli research and elite germplasm development.

## Abstract (web summary)

The ploidy level of patchouli, an aromatic plant in the Lamiaceae family, remain unclear. Here, the authors assemble a chromosome-level and haplotype-resolved genome for patchouli and reveal that it is tetraploid hybrid as well as compensated aneuploidy.

## Introduction

Patchouli (*Pogostemon cablin* (Blanco) Benth.), a species in the *Pogostemon* genus and Lamiaceae family, is a valuable medicinal and aromatic herb (Supplementary Fig. 1). Patchouli has been widely used in both traditional Chinese medicine and Ayurveda in India, which are major medical systems worldwide^1^. In China, patchouli was first documented in *Ming Yi Bie Lu* from AD 420 to 589^2^. Its shade-dried leaves were decocted with other drugs to treat cold, headache, nausea, vomiting, diarrhea, fever, dampness and to stimulate appetite^3^. In India, patchouli has been primarily used as an insect repellent in daily life and has been applied in preparations of Ayurvedic treatments such as Rasa, Guna, and Virya^3^. Moreover, patchouli has been used for the prevention of viral pandemics, such as those of SARS, bird flu, H1N1 flu and even the current COVID-19 pandemic^4,5^.

Although it is indigenous to Southeast Asia and has been used in Asian countries for many centuries, patchouli was popular in Europe until the 1840s, due to its unique aroma retained in exported Indian garments^6^. Subsequently, patchouli was widely introduced into tropical and subtropical areas of Asia due to the high demand for patchouli oil^7^. As essential oil extracted from the aerial parts of the patchouli plant, patchouli oil is currently used worldwide in the perfumery industry, including fragrances, cosmetics, toiletries, and food. The global demand for patchouli oil is >1600 tons per year^8^. Phytochemistry and pharmacological studies have shown that volatile sesquiterpene metabolites, especially patchouli alcohol, are the main phytochemical compounds of patchouli oil and have various biological activities, including antimicrobial, antiemetic, and antioxidant^9^.

Despite the broad distribution of patchouli, it is introduced mainly by stem cuttings. The flowering and seed set of patchouli are rare^8^. Although asexual reproduction has allowed the wide distribution of patchouli, it has become the main obstacle to patchouli production and improvement. Asexual reproduction decreases the genetic diversity of patchouli and increases the risk under biotic or abiotic stress, making it impossible to perform hybrid breeding in patchouli to develop new varieties. Thus, the mechanism underlying the lack of seed reproduction and an understanding of the history of its formation and evolution are urgently needed. As chromosome variants may trigger the inability of sexual reproduction, the chromosome composition and genome information of patchouli may provide some clues.

The basic chromosome number in *Pogostemon* genus is *x* = 16 or 17, while *x* = 16 is the primary, and *x* = 17 is derived by dysploidy from *x* = 16^10^. The chromosome number of patchouli is a little controversial. Although as early as 1984, Lavania has reported that the patchouli chromosome number was 32^11^, the other studies subsequently reported that the patchouli chromosome number was 64^12–17^. It seems that 2*n* = 4*x* = 64 is the most common cytotypic of patchouli. The patchouli genome was surveyed and assembled by the He et al. in 2016 and 2018^18,19^. It is a complex plant genome with high heterozygosity and large repeat content. Due to the limitations of next-generation sequencing and chromosome anchoring technology, the two assembled genomes varied dramatically (1.15 Gb vs. 1.91 Gb) and no chromosome-level sequences was obtained.

Here, we assemble a chromosome-level and haplotype-resolved reference genome for patchouli. The genome annotations demonstrate that tandem duplications contribute to the enrichment of genes involved in sesquiterpene biosynthesis. Moreover, the detailed chromosome comparisons and evolution analysis suggest that the patchouli genome is a tetraploid hybrid as well as compensated aneuploidy.

## Results

### All chromosomes of patchouli were assembled

**Fig. 1:**
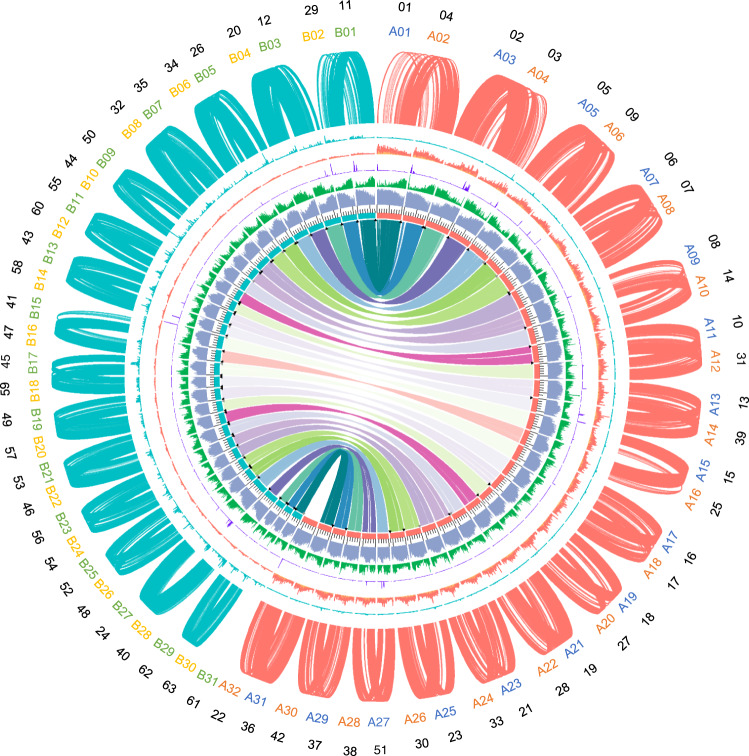
Overview of the patchouli genome. The tracks from the inner to outer regions of the circle indicate the chromosomes, density of repeat sequences, coding genes, noncoding genes, and density of enriched 13-mer sequences in the subgenomes A and B, respectively. The links inside the circle show intersubgenome syntenic gene pairs, and the links outside the circle show intrasubgenome syntenic gene pairs. The triangles on chromosomes indicate telomere regions. The regions locate at the exactly start or end of chromosomes are shown in black, while the others are shown in gray. All the density tracks are calculated by the base coverage in continuous 100 kb windows. The density ranges for repeat sequences and genes are both 0 to 1, those for noncoding genes are 0 to 0.40, those for subgenome A specific 13-mer sequences are 0 to 0.26, and those for subgenome B specific 13-mer sequences are 0 to 0.06. Chromosomes, enriched 13-mers, and syntenic gene pairs between the intrasubgenomes are colored orange for subgenome A and cyan for subgenome B. The numbers in the outermost circle are original chromosome IDs, in the penultimate circle are updated subgenome chromosome IDs; they are colored according to the mini-subgenomes they belong to. The prefix “Chr” for original chromosome IDs and the prefix “I” and “II” for updated subgenome chromosome IDs were omitted.

To clarify the genetic basis of patchouli, we attempted to assemble a high-quality patchouli genome. First, we estimated its genome size by flow cytometry using tomato (*Solanum lycopersicum*) as an internal standard^20^, and the results showed that the patchouli genome was ~1040 ± 167.35 Mb (Supplementary Fig. 2). Then, we conducted a genome survey with ~60× NovaSeq reads (Supplementary Table 1) to confirm the genome size, and determined that its heterozygosity was ~3.69% and the repeat sequence percentage was ~75.5% (Supplementary Fig. 3). To assemble the high-complexity patchouli genome, we additionally sequenced 166.6 Gb (~160×) PacBio SMRT reads with a contig N50 of 31 kb and 135.5 Gb (~135×) Hi-C reads with a valid interaction ratio of 26.63% (Supplementary Table 1). Then, genome assembly was conducted by following the pipeline shown in Supplementary Fig. 4. The final patchouli genome spanned 1.94 Gb by 1752 contigs with an N50 of 7.97 Mb, of which 827 contigs (96.45% of the sequences) were assigned to 63 chromosomes (Fig. 1). These chromosomes were named Chr01 to Chr63 from long to short.

To assess the quality of our assembly, ultralong Oxford Nanopore Technology (ONT) sequencing was performed. As 35,101 ONT reads >100 kb were aligned to the 63 chromosomes (Supplementary Table 1), 96.05% (33,714) of them mapped to a single chromosome with >90% of their own length, covering 87.45% of the total chromosomal bases. Furthermore, 99.42% of the NovaSeq reads used for the genome survey were aligned to the assembled genome properly. The low degree of interaction between chromosomes in the Hi-C contact matrix also indicated that there was no obvious assembly error in our genome (Supplementary Fig. 5). The BUSCO analysis indicated that the genome’s completeness reached 95.4%. Moreover, telomere repeat regions were explored in our genome as they represent the end of the linear chromosome. In total, 77 telomere repeat regions were detected, of these, 56 were distributed on both ends of 28 chromosomes and the remaining regions were distributed on either end of 21 chromosomes (Fig. 1). Taken together, this evidence indicated the high completeness and accuracy of our patchouli genome.

The length of our assembled genome was nearly twice that predicted by the flow cytometry and genome survey, indicating that our assembly could be haplotype-resolved. However, only 63 chromosomes were assembled, which is inconsistent with previous studies^13,14^. Our chromosome counting also showed that the patchouli somatic cells contain 64 chromosomes (Supplementary Fig. 6). We investigated the sequence depth and GC content of continuous 10 kb regions in the assembled genome by NovaSeq read remapping and found that although most of the regions had a depth of ~27×, some regions had a depth of ~55× (Supplementary Fig. 7). This result inspired us to speculate that one chromosome in our assembled genome may represent the sequence of two homologous chromosomes in patchouli somatic cells. The sequence depth of each chromosome was analyzed and we found that the depth of Chr22 was twice that of the other chromosomes (Supplementary Fig. 8). Moreover, when mapping original ultralong ONT reads back to the assembled genome, we found the sequence depth of Chr22 was also nearly twice that of the other chromosomes (Supplementary Fig. 9b), while its average map quality was almost the same as those of other chromosomes (Supplementary Fig. 9a). We further investigated the Chr22 assembly in details. We called SNPs using 136,123,184 reads from a patchouli accession from Indonesia and found that the SNP density and heterozygosity percentage of Chr22 were normal compared with those of other chromosomes (Supplementary Data 1). This evidence indicates that our assembled 63 chromosomes contain the sequence information of 64 chromosomes in patchouli somatic cells as Chr22 represents two homologous chromosomes.

Considering the patchouli plant we sequenced have undergone tissue culture, we want to exclude the possibility of somaclonal mutation which could alter chromosome composition. We sequenced the whole genome of a patchouli plant from Yangchun, Guangdong Province, which was the original place of our sequenced patchouli plant. The read depth distribution of the Yangchun patchouli plant is similar with the results of our sequenced plant (Supplementary Fig. 10 vs. Supplementary Fig. 8), which suggest no somaclonal mutation in the patchouli plant we sequenced. Furthermore, to check whether the patchouli plant we sequenced was representative, a specific-locus amplified fragment sequencing (SLAF-seq) dataset was analyzed. The dataset contains 22 patchouli accessions collected from eight localities in China, one locality in Vietnam, and ten localities in Indonesia^21^. The genome size of these patchouli accessions was 1226 Mb^21^, which is consistent with the result of our sequenced patchouli plant. The SLAF-seq reads of each accession were aligned to our assembled genome. To avoid nonunique mapping, only reads with a map quality >30 were counted. As expected, many more mapped reads were located on Chr22 than on the other chromosomes (Supplementary Data 2). These results indicate that the patchouli genome we assembled was representative.

### Genome annotation revealed tandemly duplicated genes in the sesquiterpene biosynthetic pathway

Ab initio prediction, homolog-based searches and transcript evidence gathered from multiple RNA-seq datasets were combined to annotate protein-coding genes. In total, 109,696 genes were predicted in the patchouli genome with an average transcript length, coding sequence length and exon number of 1662 bp, 1279 bp, and 6.0, respectively (Fig. 1). Although the gene number of patchouli was much higher than that of the other species of Lamiales, it was equivalent (109,611 vs. 110,850) to that observed in a previous patchouli study^18^, and the transcript statistics of patchouli were similar to those of the other species of Lamiales (Supplementary Table 2). The vast majority of gene models (96.08%) were supported by the RNA-seq datasets, and 77.19% matched at least one public protein database. The completeness of the gene repertoire was assessed by Benchmarking Universal Single-Copy Orthologs (BUSCO) (v.4.0.6). For the 2124 core single-copy orthologous genes from the eudicotyledon lineage, 98.72% were complete in our annotation. Among them, 2067 were duplicated and 1228 were duplicated four times, suggesting that the patchouli genome may have undergone two rounds of whole-genome duplications during its evolution. In addition, noncoding genes were annotated, including 1748 tRNAs, 3609 rRNAs, 1244 miRNAs, 564 snRNAs, and 8427 snoRNAs (Fig. 1).

Protein-coding genes involved in the “plant-pathogen interaction”, “DNA replication” and “sesquiterpenoid and triterpenoid biosynthesis” pathways were enriched in patchouli more than five times when compared to the other 7 species of Lamiales (Supplementary Fig. 11). As sesquiterpenes are the major bioactive components of patchouli, we further dissected the terpene biosynthesis pathway. Enzyme-coding genes were explored based on the genome annotation. In total, 38 genes encoding 6 enzymes in the mevalonate (MVA) pathway, 45 genes encoding 7 enzymes in the methylerythritol 4-phosphate (MEP) pathway, four genes encoding isopentenyl diphosphate isomerase (IDI), four genes encoding geranyl diphosphate synthase (GPPS), four genes encoding farnesyl diphosphate synthase (FPPS), and 8 genes encoding geranylgeranyl diphosphate synthase (GGPPS) were identified (Supplementary Fig. 12). The expression patterns of the genes encoding homologous enzymes were similar, excluding those encoding HMGR in the MVA pathway and DXS in the MEP pathway. The genes in the MVA pathway showed higher expression in the root (S2), stem (S3) and leaf (S6) tissues and could be induced by treatment with ethanol (S15) or methyl jasmonate (MeJA) and ethanol simultaneously (S14) (Supplementary Fig. 12). Consistent with the BUSCO analysis, 14 of these 17 enzymes were encoded by homologous genes with a copy number of 4 or a multiple of 4, while the remaining three enzymes were encoded by homologous genes that were tandemly duplicated. Three *HMGR* genes and three *HDR* genes were tandemly duplicated on Chr14, two *DXS* genes were tandemly duplicated on Chr36, and two *DXS* genes were tandemly duplicated on Chr22.

**Fig. 2:**
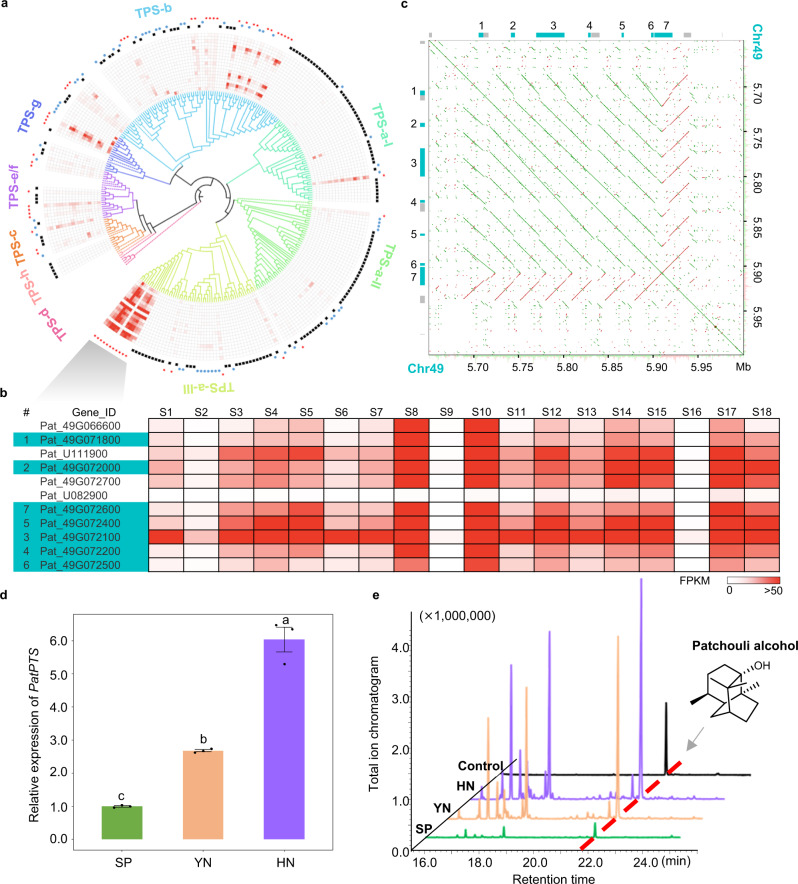
Tandemly duplicated genes in the sesquiterpene biosynthetic pathway. **a** Phylogenetic tree and expression pattern of 266 TPS genes in the patchouli genome. Solid lines in the phylogenetic tree represent patchouli genes, dashed lines represent genes from other species with clear subfamily definitions. The gene is denoted by a black square when its total FPKM is <1 in 18 RNA-seq datasets, by a blue square when its FPKM is >1 in 1–5 RNA-seq datasets and by a red star when its FPKM is >1 in >6 RNA-seq datasets. **b** Magnified view for part of the PTS-a-III group with high expression. The seven tandemly duplicated PatPTS genes on Chr49 are highlighted by cyan. **c** Dot-plot analysis of sequences from 5.65 Mb to 6.00 Mb on Chr49. Boxes indicate gene locations, and cyan boxes indicate genes numbered in Fig. 2b. Green and red dots represent forward and reverse alignments, respectively. **d** Total relative expression level of seven PatPTS genes in mature leaves of the SP, YN, and HN accessions. The values of three replications are shown for each accession and presented as mean values ± SEM. The different letters above each accession indicate significant differences by One-way ANOVA. LSD test and Bonferroni’s adjustment were performed for multiple comparison. **e** Relative abundance of patchouli alcohol in mature leaves of the SP, YN, and HN accessions. Source data are provided as a Source Data file.

Terpene synthase (TPS) is the most important key enzyme in terpene biosynthesis. We annotated 266 full-length TPS genes in our patchouli genome, which was much more than that predicted in previous studies^18^. According to the whole phylogenetic structure and location of 28 known TPS genes in other species, the patchouli TPSs were classified into five families, namely, TPS-a (144), TPS-b (79), TPS-c (12), TPS-e/f (17) and TPS-g (14). The TPS-a members were further classified into three subfamilies, namely, TPS-a-I, TPS-a-II, and TPS-a-III (Fig. 2a). TPS-a mainly corresponds to sesquiterpene synthase (sesTPS), TPS-b and TPS-g mainly correspond to monoterpene synthase (monoTPS), and TPS-c and TPS-e/f mainly correspond to diterpene synthase (diTPS). Among the 266 TPS genes, 255 were located on 45 chromosomes. Of these, 172 were located on 46 tandemly duplicated gene clusters from 23 chromosomes (Supplementary Fig. 13). The tandem duplication rate of the TPS genes was 67.45%, which was much higher than that of the whole genome (5.44%). More than 60% of the TPS-a and TPS-b genes were tandemly duplicated, and this proportion was much higher for TPS-a-I as 82.93% (Supplementary Table 3). However, 56.77% (151/266) of the TPS genes were almost not expressed (FPKM < 1) in all our collected patchouli RNA-seq datasets, and only 21.05% (56/266) were expressed in >5 samples. In particular, 87.50% (126/144) of the genes in the TPS-a-I subfamily were almost not expressed (Fig. 2a). Although TPS genes have undergone wide expansion by tandem duplication, these expansions may not contribute to terpene biosynthesis.

This hypothesis was contradicted by a tandemly duplicated gene cluster in the TPS-a-III subfamily containing seven genes that were highly and widely expressed in the RNA-seq datasets (Fig. 2a, b). A dot-plot analysis of Chr49 from 5.65 Mb to 6.00 Mb was performed. As shown in Fig. 2c, these genes were derived from tandem duplications of a region of ~30 kb, and seven forward tandem duplications and one reverse duplication were included. Containing sesquiterpene synthase-specific motifs such as DDXXD, NSE/DTE, and R(X)_9_ W^22^, the seven genes were annotated as patchouli alcohol synthase (*PatPTS*). These genes had high protein sequence similarities (97.74%–100%) with each other (Supplementary Table 4), and a representative one (*Pat_49G072100*) can catalyze the conversion from farnesyl diphosphate to sesquiterpenes, similar to the reported *PatPTS* gene (https://www.ncbi.nlm.nih.gov/nuccore/AY508730.1/)^23^ in which patchouli alcohol was included (Supplementary Fig. 14). As the primary bioactive component of patchouli, the content of patchouli alcohol is the most important trait of patchouli. The positive correlation between the expression level of *PatPTS* and the content of patchouli alcohol was confirmed by using different patchouli accessions. While YN and HN had higher expression levels of *PatPTSs* (Fig. 2d), their relative abundances of patchouli alcohol were consistent (Fig. 2e, Supplementary Table 5). Therefore, as all seven tandem duplicated *PatPTSs* were highly expressed, it is possible that the tandem duplication of *PatPTS* might contribute to the patchouli alcohol biosynthesis of patchouli.

### The assembled patchouli genome can be phased into A and B subgenomes

**Fig. 3:**
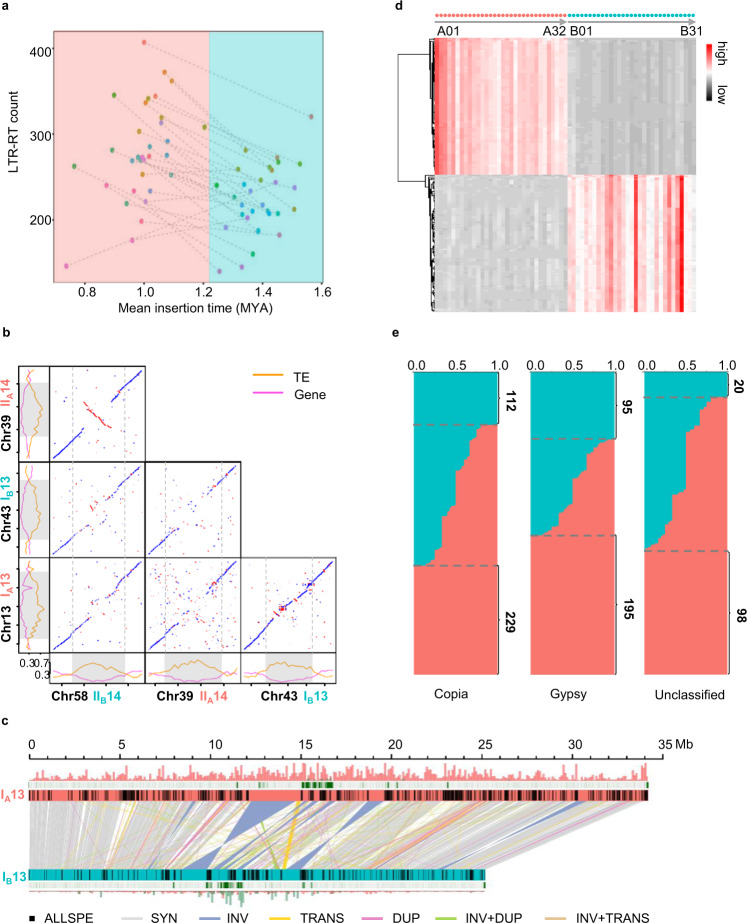
The patchouli genome can be phased into two subgenomes. **a** Distribution of the count and mean insertion time of full-length LTR-RTs for each chromosome. The background is divided according to the subgenomes to which those chromosomes belong, with orange for subgenome A (32 chromosomes) and cyan for subgenome B (31 chromosomes). MYA is an abbreviation for million years ago. **b** Dot-plot visualization of collinearity between chromosomes Chr13 (I_A_13), Chr39 (II_A_14), Chr43 (I_B_13), and Chr58 (II_B_14) in relation to the distribution of the gene and repeat sequence density (left and bottom panel boxes; pink and yellow lines, respectively). Syntenic regions >5 kb are shown. Blue and red dots represent the forward and reverse alignment, respectively. **c** Comparisons of interchromosome homoeologous chromosome I_A_13 and I_B_13. Only regions >5 kb are shown. The heatmap tracks for each chromosome represent the density of tandem repeats and the histogram tracks represent the density of enrichment 13-mer sequence. Detailed information are shown in Supplementary Fig. 18. **d** Clustering of enriched 13-mer sequences for their counts in each chromosome. Only the top 100 enriched 13-mers for subgenomes A and B are shown. **e** The proportion of subgenome belonging for elements in each LTR-RT subfamily that contain more than one element. The *Y*-axis represents each subfamily, while the *X*-axis is the percentage composition of element locations (subgenomes A or B). The percentage composition for subgenome A is colored orange, and for subgenome B is colored cyan. Source data are provided as a Source Data file.

The annotated genes were embedded within a sea of transposable element (TE) relicts and other repetitive sequences, which accounted for 65.94% of the patchouli genome (Supplementary Table 6, Fig. 1). We detected full-length TEs with solid structures and estimated their insertion times (Supplementary Fig. 15), including 6271 Copia, 5396 Gypsy, and 4204 other long terminal repeat-retrotransposons (LTR-RTs) belonging to an unknown superfamily (Unclassified). Interestingly, we observed that the 63 chromosomes we assembled could be divided into two groups according to the count and insertion time of their full-length LTR-RTs. The larger group contained 32 chromosomes with more LTR-RTs and later insertion times, while the smaller group contained 31 chromosomes with fewer LTR-RTs and earlier insertion times (Fig. 3a). The chromosomes in the larger group were also significantly longer than those in the smaller group (Supplementary Fig. 16). Similarly, the LTR assembly index (LAI), a parameter used to evaluate genome assembly continuity^24^, was much greater for chromosomes in the larger group than for those in the smaller group (Supplementary Table 7). The above evidence suggests that the patchouli genome could be divided into two subgenomes. Therefore, we designated 32 chromosomes in the larger group as subgenome A and 31 chromosomes in the smaller group as subgenome B.

To explore the chromosome relationship between and within subgenomes A and B, we conducted a collinearity analysis for chromosome sequences. For all 31 chromosomes in the B subgenome, there was an obvious collinearity chromosome in subgenome A, i.e., Chr13 vs. Chr43 and Chr39 vs Chr58. In addition, high collinearity was also detected between chromosomes within the subgenome, for example, Chr13 vs. Chr39 and Chr43 vs. Chr58 (Fig. 3b, Supplementary Fig. 17). The collinearity relationship between subgenomes A and B indicated that they might have evolved from a same ancestor, and the collinearity relationship within each subgenome indicated that they both had undergone a round of chromosome doubling. To clearly represent the collinearity relationship, we renamed the chromosome IDs. The collinear chromosomes between subgenomes A and B (intersubgenome homoeologous chromosomes) were renamed with the same ID number and prefix A or B. The collinear chromosomes within subgenome A or B (intrasubgenome homoeologous chromosomes) were renamed with a continuous ID number and prefix I or II (Supplementary Data 3). According to the nomenclature rule, the chromosomes in subgenome A were designated I_A_01, II_A_02, I_A_03, II_A_04 to I_A_31, while their homoeologous chromosomes in subgenome B were designated I_B_01, II_B_02, I_B_03, II_B_04 to I_B_31. The remaining Chr22 in subgenome A did not have a homoeologous chromosome in subgenome B, and we renamed it II_A_32. A clear syntenic relationship between the chromosomes was also observed by an analysis at the gene level as shown in Fig. 1.

Although the collinearity between the intersubgenome homoeologous chromosomes was clear, the length between them was significant difference. Therefore, we compared these chromosomes in-depth. Among 31 comparison pairs, only 49.50% of the sequences were synteny (SYN), while 19.55% of the sequences were only contained by specific subgenomes (ALLSPE) (Supplementary Table 8). In addition, a large number of structural variations were detected, including 657 inversion events (INV), 3176 translocation events (TRANS), 18,765 duplication loss/gain events (DUP), 2802 events involving not only translocation but also inversion (INVTR) and 15,693 events involving not only duplication loss/gain but also inversion (INVDP) (Fig. 3c, Supplementary Fig. 18).

In addition to the structural variations, subgenome-enriched 13-bp sequences (13-mers) were explored in our genome, which can provide robust markers of subgenome ancestry^25^. We detected 25,919 and 163 enriched 13-mers in subgenomes A and B, respectively (Supplementary Fig. 19, Fig. 1). The top 100 of each subgenome are clustered in Fig. 3d. To test the sensitivity of this method, we performed permutation tests by exchanging the corresponding subgenome of chromosomes in one homoeologous pair and then detected the enriched 13-mers again. For each homoeologous pair, fewer than five 13-mers were detected as being enriched in subgenome A, and no 13-mers were detected as being enriched in subgenome B (Supplementary Table 9). As the permutation test results showed a dramatic contrast with the real haplotype results, we believe that we phased the patchouli genome correctly and that specific sequences were enriched in subgenomes A and B. Evidence from the LTR-RT analysis further supported this conclusion. Full-length LTR-RTs in three superfamilies (Copia, Gypsy, and Unclassified) were classified into subfamilies (Supplementary Fig. 15). The subfamilies that contained more than one element and all these elements located in only subgenome A or subgenome B were defined as “subgenome specific”. In total, 229 Copia subfamilies, 195 Gypsy subfamilies, and 98 Unclassified subfamilies were detected as being subgenome A specific, while 112 Copia subfamilies, 95 Gypsy subfamilies, and 20 Unclassified subfamilies were detected as being subgenome B specific (Fig. 3e). In each LTR-RT superfamily, the subgenome specific subfamilies contained fewer elements than the nonspecific subfamilies, and the insertion time of elements belonging to subgenome specific subfamilies was much later than that of elements belonging to nonspecific subfamilies. Most LTR-RT elements belonging to subgenome specific subfamilies were inserted into the subgenome later than 1 MYA (Supplementary Fig. 20).

### Subgenomes A and B diverged mainly by LTR-RT insertion in subgenome A

**Fig. 4:**
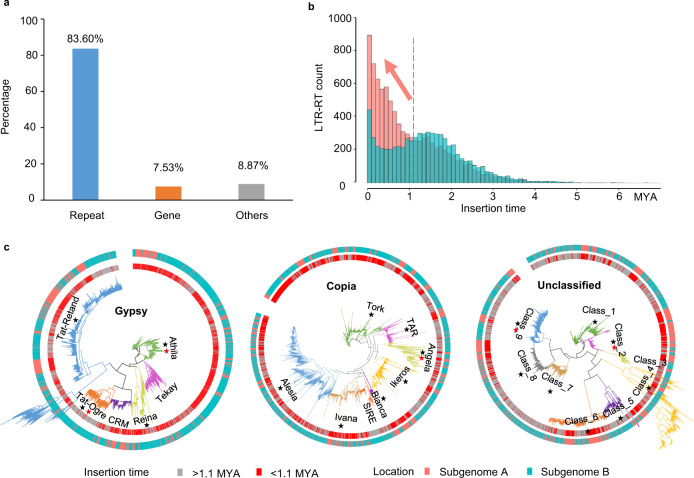
The subgenomes A and B diverged mainly by insertion of long terminal repeat-retrotransposons (LTR-RT) on subgenome A. **a** Composition of different genomic features in the patchouli genome. **b** Histogram of the insertion time of full-length LTR-RTs in subgenomes A and B. **c** Lineage classification of LTR-RT superfamilies. The subgenome location and insertion time of Copia, Gypsy, and Unclassified LTR-RTs are indicated by the outer and inner circles, respectively. Red stars and black stars denote the lineages whose length and count expanded in subgenome A after 1.1 million years ago (MYA), respectively.

As intersubgenome homoeologous chromosomes have high collinearity and obvious sequence divergence, it is interesting to understand how they diverged. We calculated and compared the gene and repeat lengths of each chromosome pair (Supplementary Table 10). For the 310 Mb size differences between subgenomes A and B, 83.60% was attributed by variation in repeat regions (Fig. 4a). Therefore, the lengths of the LTR-RT superfamilies and DNA transposons were further compared, as they were the major components of the repeat sequences. Subgenome A contained a significantly greater number of Copia, Gypsy, Unclassified LTR-RTs, and DNA transposon relicts than subgenome B (Supplementary Table 11). The distribution of the LTR-RT insertion time showed a rapid and continuous expansion of LTR-RTs in subgenome A after 1.1 MYA (Fig. 4b). Then, we conducted the same analyses of the three LTR-RT superfamilies (Supplementary Fig. 21). Although their distribution patterns slightly differed from each other, the recent expansion of subgenome A was explicit in all of them.

Additional evidence was obtained to confirm the LTR-RT insertion. We selected 93.85% Gypsy (5064/5396), 90.29% (5662/6271) Copia, and 75.02% (3154/4204) Unclassified full-length LTR-RTs containing PF00078, PF07727, and PF03732 domains to construct phylogenetic trees and classified them into different lineages according to the Viridiplantae_v3.0 database. The number and length of LTR elements inserted before and after 1.1 MYAs were compared between subgenomes A and B in each lineage. The counts of all LTR-RT lineages significantly expanded in subgenome A after 1.1 MYA, except for CRM and Tkeay in Gypsy, SIRE in Copia and Class_3 in the Unclassified superfamily (Fig. 4c, Supplementary Table 12). Moreover, Athila and Tat_Ogre in Gypsy, Angela in Copia, and Class_9, Class_6 and Class_2 in the Unclassified superfamily showed larger differences in the subgenome lengths after 1.1 MYA and before 1.1 MYA (Supplementary Fig. 22). Based on these results, we can speculate that the burst of universal LTR-RTs in subgenome A was the major contributor to the divergence between subgenomes A and B.

### No significant dominance between subgenomes A and B

How subgenomes A and B function in the patchouli genome remains unclear. Did they undergo asymmetric gene loss, biased gene expression or specific nature selection, as observed in some allopolyploid plants^26,27^. To answer these questions, comparisons of the gene constitution, expression, and genetic diversity between subgenomes A and B were performed.

**Fig. 5:**
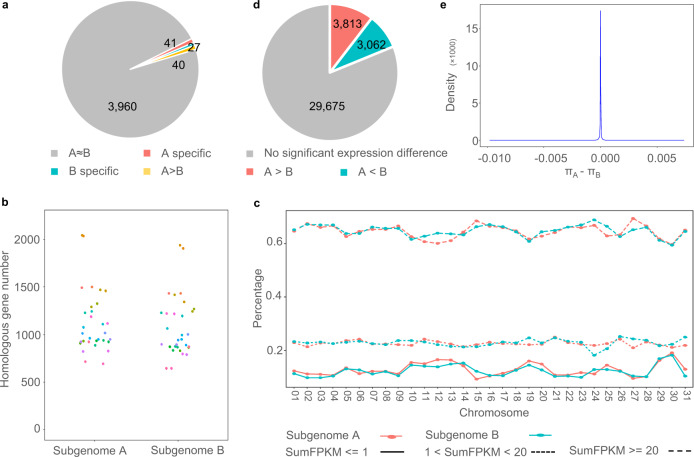
Comparison of dominance between subgenomes A and B. **a** The counts of genes containing each Pfam domain are compared between subgenomes A and B. The Pfam domains are classified into “A ≈ B” (contained gene counts are not significantly different), “A specific” (only contained by subgenome A), “B specific” (only contained by subgenome B), and “A > B” (contained by more genes in subgenome A). **b** Comparison of chromosomal homologous gene numbers between subgenomes A and B. *Scutellaria barbata* was used as an outgroup. Wilcoxon test was performed with two-sided alternative hypothesis. **c** Comparison of *Scutellaria barbata* homologous gene expression between subgenomes A and B. Genes were classified into three groups according to their total expression value in 18 RNA-seq datasets. The percentage of each group was compared for each chromosome. FPKM is an abbreviation for Fragments Per Kilobase Million. **d** Comparisons of the expression of genes in each intersubgenome syntenic gene pair. *T*-test were performed with two-sided alternative hypothesis. Gene pairs are classified into “No significant expression difference”, “A > B” (genes from subgenome A highly expressed than corresponding genes from subgenome B), and “A < B” (genes from subgenome B highly expressed than corresponding genes from subgenome A). **e** Kernel density plot for the differences in genetic diversity between genes belonging to intersubgenome syntenic gene pairs. Source data are provided as a Source Data file.

The protein domain is a key indicator of gene function. If gene loss occurred largely in a specific subgenome, the count of genes containing corresponding protein domains would decrease. Therefore, we first compared the counts of genes belonging to each Pfam domain between subgenomes A and B. To eliminate the influence of the asymmetric II_A_32 chromosome, genes located on this chromosome were excluded. Among the 105,700 genes located on 62 chromosomes, 4068 Pfam domains were present in 80,265 (74.79%) genes. Using the total gene number of each subgenome as background, the Pfam domains were considered to have significantly different gene counts between subgenomes A and B when *p* was <0.05 in the Fisher’s exact test. As shown in Fig. 5a, 97.32% (3960/4068) of the Pfam domains were contained by almost the same number of genes in subgenomes A and B, indicating that their gene function did not change dramatically. On the other hand, using *Scutellaria barbata* as an outgroup, the *Scutellaria barbata* homologous genes were searched in the patchouli genome and compared between subgenome A and subgenome B. To diminish the effect of TE insertion, genes containing TE-related Pfam domains^28^ were excluded from this analysis. As shown in Fig. 5b, the counts of the homologous genes retained in the two subgenomes were not significantly different (*p* = 0.304). This evidence further confirms that no large gene loss occurred in any subgenome after their divergence.

The expression of *Scutellaria barbata* homologous genes in patchouli was explored. The genes were classified into three different expression groups according to their total FPKMs in the 18 RNA-seq datasets. The percentage of homologous genes belonging to different expression groups was compared between intersubgenome homoeologous chromosomes. As the percentages of the two subgenomes did not show persistent differences in any expression group (Fig. 5c), we speculated that the gene expression also did not change dramatically between subgenomes A and B. In addition, paired *t*-tests were performed to analyze the expression of each intersubgenome homologous gene pair. A gene pair was identified as significantly differentially expressed when its two genes’ FPKMs had a fold change >2 and a *p*-value < 0.05 in 18 RNA-seq datasets. Among all 36,550 homologous gene pairs, most (81.19%) exhibited similar expression in subgenomes A and B, while only 10.43% (3813/36,550) and 8.37% (3062/36,550) were significantly highly expressed in subgenomes A and B respectively (Fig. 5d). Furthermore, these highly expressed genes in subgenomes A and B were both enriched in pathways related to secondary metabolism, DNA replication, and plant-pathogen interactions (Supplementary Fig. 23), indicating that the differential expression in homologous gene pairs might be random. Therefore, there was no clear gene expression bias in subgenome A or B.

Hybridization is an important evolutionary force. It is interesting to compare the natural selection pressures that the two subgenomes experienced after hybridization. We sequenced five other patchouli accessions that are widely planted in China and Southeast Asia and detected 130,981 and 159,937 high-quality SNPs in subgenomes A and B, respectively. The genetic diversity (*π*) was calculated in continuous 100 kb windows of each chromosome. Although subgenome A had a lower average *π*-value than subgenome B (4.92195E-05 vs. 8.74616E-05), their mean *π*-values were similar (2.46E-05 vs. 2.32E-05). The high average *π*-value in subgenome B was caused by high *π*-values in some continuous regions, as shown in Supplementary Fig. 24. The genetic diversity was also compared within intersubgenome homologous gene pairs. Most pairs (83.46%) had the same *π*-value (*π*_A_ − *π*_B_ = 0), and most of other homologous gene pairs had *π*-values with a very small difference (*π*_A_ − *π*_B_ ≈ 0) (Fig. 5e). Therefore, we hypothesized that the two subgenomes in the patchouli genome had experienced the same selection pressure.

### Evolutionary scenario of the patchouli genome

**Fig. 6:**
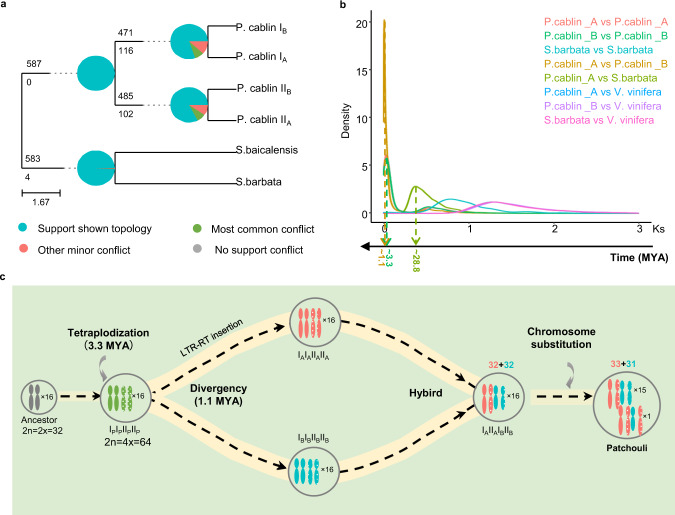
Evolution of patchouli genome. **a** ASTRAL-III tree with summary of conflicting and concordant gene trees produced with Phyparts (v0.0.1). For each branch, the top number indicates the number of gene trees concordant with the species tree at that node, and the bottom number indicates the number of gene trees in conflict with that clade in the species tree. The pie charts at each node present the proportion of gene trees support each type. **b** Kernel density plots for corrected Ks between genomes among patchouli subgenome A, patchouli subgenome B, *Scutellaria barbata*, and *Vitis vinifera* genomes. **c** Evolutionary scenario of patchouli genome. Solid line represents chromosomes from I group, mosaic line represents chromosomes from II group. Orange line represent chromosomes from subgenome A and cyan line represent chromosomes from subgenome B. MYA is an abbreviation for million years ago.

The haplotype-resolved patchouli genome enabled us to trace its phylogenetic position and evolutionary history. Combing with other 7 Lamiales species, *Solanum lycopersicum*, *Arabidopsis thaliana*, and *Oryza sativa*, 587 single-copy nuclear genes were identified to construct maximum likelihood phylogenetic tree (Supplementary Fig. 25). The genus closest to *Pogostemon* (patchouli) was *Scutellaria* (*Scutellaria barbata* and *Scutellaria baicalensis*) and they may have diverged from 28.8 MYA. Chromosomes in the patchouli genome were divided into four mini-subgenomes I_A_, II_A_, I_B_, and II_B_. Phylogenetic relationship between them were analyzed by constructing the ASTRAL-III tree with summary of conflicting and concordant gene trees. As Fig. 6a shown, the I_A_ mini-subgenome was evolutionarily closer to I_B_ than to II_A_, and II_A_ was closer to II_B_ than to I_A_. This topology was also supported by most of individual gene trees. Therefore, the divergence between groups I and II may occurred earlier than the divergence between subgenomes A and B.

To confirm this hypothesis, the structural variations between intrasubgenome homoeologous chromosomes (such as, I_A_01 vs. II_A_02 and I_B_01 vs. II_B_02, Supplementary Fig. 26) were detected and compared with the results of the intersubgenome homoeologous chromosomes (such as, I_A_01 vs. I_B_01 and II_A_02 vs. II_B_02, Supplementary Fig. 18). Consistent with our expectation, structural variations occurred more frequently between intrasubgenome homoeologous chromosomes than intersubgenome homoeologous chromosomes. The detailed statistics also showed that only 21.48% of the sequences were syntenic between the intrasubgenome homoeologous chromosomes, while this value was 49.50% between the intersubgenome homoeologous chromosomes (Supplementary Table 13 vs. Supplementary Table 8). In addition, the hypothesis was supported by the higher percentage of syntenic genes and sequence collinearity between the intersubgenome homoeologous chromosomes than between the intrasubgenome homoeologous chromosomes (Supplementary Fig. 27, Supplementary Fig. 17). More deeply, we compared the structural variations that occurred between homeologous chromosome pairs within subgenomes A and B. As shown in Supplementary Fig. 28, the length percentage of the structural variation types, such as duplication (DUP), inversion and duplication (INVDP), inversion and translocation (INVTR) and translocation (TRANS), were significant higher within subgenome A than within subgenome B. These results indicate that the considerable divergence between the subgenomes was induced by structural variations mainly occurring in subgeome A, which is consistent with our analysis based on LTR-RT insertions.

It is important to determine whether the chromosome doubling event occurred simultaneously within subgenomes A and B. The density of synonymous substitution rates (Ks) and the distance-transversion rate at fourfold degenerate sites (4DTv) of syntenic gene pairs within and between patchouli subgenome A, patchouli subgenome B, *Scutellaria barbata* and *Vitis vinifera* were calculated (Supplementary Fig. 29). As the peaks of the Ks and 4DTv distribution patterns within subgenomes A and B were almost the same, chromosome doubling within them may have occurred simultaneously. In addition, the Ks and 4DTv peaks between subgenomes A and B were earlier than those with subgenomes A and B, respectively, which is consistent with our previous conclusion that chromosome doubling events within the subgenomes occurred prior to the divergence between subgenomes A and B. As divergence in evolutionary rates may jeopardize efforts to date evolutionary events^29^, we performed a correction to the Ks distribution by using the known evolutionary history as a reference. Assuming that the divergence between patchouli and *Scutellaria barbata* was about 28.8 MYA, we estimated that the chromosome doubling within the subgenomes occurred ~3.3 MYA and that sugenomes A and B diverged from ~1.1 MYA (Fig. 6b).

We integrated all results from the genome comparisons and evolutionary analysis as follows. The 63 patchouli chromosomes we assembled contain sequence information of 64 chromosomes in patchouli somatic cells. These chromosomes could phase into subgenomes A and B, as the original II_B_32 was substituted by an extra copy of II_A_32. Homoeologous chromosome pairs existed within and between the subgenomes. Chromosome doublings occurred simultaneously within the subgenomes (~3.3 MYA) and occurred earlier than intersubgenome divergence (~1.1 MYA), which was mainly induced by special LRT-RT burst in subgenome A. In addition, no significant dominance was detected between subgenomes A and B.

Based on these results, we propose a scenario of patchouli evolution (Fig. 6c). An ancient diploid plant from the *Pogostemon* genus (2*n* = 2*x* = 32) underwent a round of chromosome doubling at ~3.3 MYA, forming a tetraploid species (I_P_I_P_II_P_II_P_, 2*n* = 4*x* = 64). Then, this species diverged into two different species, I_A_I_A_II_A_II_A_ and I_B_I_B_II_B_II_B_, mainly by the burst of LTR-RT in I_A_I_A_II_A_II_A_ after 1.1 MYA. Patchouli originated from the intraspecific hybrid between I_A_I_A_II_A_II_A_ and I_B_I_B_II_B_II_B_, while a specific chromosome substitution occurred during this process.

## Discussion

With the rapid development of both sequencing technologies and assembly algorithms, it is much easier to assemble chromosome-scale and haplotype-resolved plant genomes, even for autopolyploid and allopolyploid species^30–32^. However, the patchouli genome we assembled is very special, which is tetraploid hybrid with compensated aneuploidy.

Originating from hybridization between two tetraploid congeners in *Pogostemon*, the homoploid hybrid origin of patchouli is intriguing. In contrast to allopolyploid hybrid speciation, which involves the full duplication of a hybrid genome, homoploid hybrid speciation is the origin of a hybrid species without a change in the chromosome number^33^. Although there are fewer cases of homoploid hybrid speciation than allopolyploid speciation, it has been reported in animals, fungi, and plants^34,35^. The detection of homoploid hybrid speciation is difficult due to the absence of easily observed changes in chromosome number^36^. Only about 20 well-established homoploid hybrid species have been detected in plants^37^. In this study, we speculated that the patchouli is a homoploid hybrid based on the genome assembly and comparisons, which provide a way for the determination of homoploid hybrid species.

The high levels of heterozygosity introduced by hybridization could lead to a shift to asexual reproduction^38^, which can enable the long-term maintenance of heterozygosity in ways similar to allopolyploid. Therefore, homoploid hybrid speciation may be the basic cause of patchouli asexual reproduction. As subgenomes A and B diverged from same ancestor, and higher collinearity as well as closer phylogenetic relationships were detected between them, intersubgenome homoeologous chromosomes would undergo pairing and synapsis during meiosis. However, significant chromosome length differences, complex structural variations, and distinct sequence characteristics such as different TE insertion patterns and subgenome specific sequences (13-mers and LTR-RT subfamilies) existed simultaneously, indicating that intersubgenome homoeologous chromosomes may unlikely to pair efficiently during meiosis. We observed that inversions occurred commonly between the centromeric regions of intersubgenome homeologous chromosomes (Supplementary Fig. 18). As centromeres are an important chromosomal domains responsible for correct chromosome segregation during cell division, structural variations between them could likely lead to meiotic abnormalities, such as bridges and fragments^39,40^.

More evidence is needed to validate this hypothesis in the future. Some meiotic chromosome spreads could be very interesting to observe during patchouli flowering. When confirmed, new octoploid patchouli germplasm (2*n* = 8*x* = 128, I_A_I_A_II_A_II_A_I_B_I_B_II_B_II_B_) could be created by manually inducing whole genome duplication in present hybrid tetraploid patchouli accession. When each chromosome can pair with its homologous chromosome during meiosis, the new patchouli germplasm could reproduce by sexual system. Thus, the introduction and elite germplasm breed of patchouli could be prompted.

The patchouli genome is compensated aneuploidy, in which the chromosome numbers varied within parental subgenomes (an extra copy of chromosome II_A_32 substituted the original chromosome II_B_32), but the total chromosome number remained unchanged (2*n* = 4*x* = 64). The extra copy of chromosome II_A_32 was validated by the NovaSeq remapping depth, SNP density, and ultralong ONT reads. Furthermore, to the best of our knowledge, this chromosome substitution may exist in other patchouli accessions around the world. As a type of chromosome variation after polyploidy, compensated aneuploidy has been used extensively in wheat breeding^41^, and reported in both experimental and natural neoallopolyploids^42,43^. Specifically, for *Tragopogon miscellus* (Asteraceae), a recently formed natural allopolyploid species, among the 76 individuals checked, 56.58% were compensated aneuploidy^42^. The compensated aneuploidy of patchouli is also an indicator of its hybrid history.

Subgenome dominance is common in hybrid and polyploid species. After the merging of divergent parental subgenomes, asymmetric gene loss (or conversely, retention) and biased gene expression between subgenomes was induced by subgenome conflict^44–46^. In contrast to many other allopolyploids, no large-scale gene loss, expression dominance, or natural selection acceleration was detected between patchouli subgenomes A and B. The ‘freezing’ of subgenomes A and B may have been induced by the lack of homoeologous chromosome pairing and recombination. The resulting asexual reproduction maintains the original hybrid genome to date.

Since we provided a possible evolutionary scenario for patchouli, the authenticity of this model has yet to be tested. The most direct way is to identify the intermediate progenitor species I_P_I_P_II_P_II_P_, I_A_I_A_II_A_II_A_, and I_B_I_B_II_B_II_B_. Although we attempted to explore the phylogenetic relationship between patchouli and other congeners in *Pogostemon*, by searching related literatures^47–49^, constructing maximum likelihood tree based on transcriptome data and remapping whole genome sequence reads to the assembled genome^50,51^ (Supplementary Fig. 30, Supplementary Table 14), there was no possible progenitor was found. As we do not know whether I_P_I_P_II_P_II_P_ is extant, it is impossible for us to compare the identity of the original I and II genomes directly. Therefore, we are uncertain of the process of tetraploidization in the early stage of our model, which could be induced by WGD (autopolyploid) or hybridization (allopolyploid).

The hybrid and compensated aneuploidy patchouli genome presented here provide a comprehensive map of sesquiterpene biosynthesis and indicate a special patchouli evolution scenario involving tetraploidization, TE expansion, intraspecific hybridization, and chromosome substitution. This high-quality reference genome could offer unprecedented genomic resources for fundamental patchouli research and germplasm resource utilization and development in the future.

## Methods

### Plant material and genome sequencing

A mature *Pogostemon cablin* (Blanco) Benth. branch was collected from the medicinal botanical garden (Yaowang Mountain) at Guangzhou University of Chinese Medicine, and then tissue cultured for propagation. Young leaves from seedlings were used for the genome sequencing. The original patchouli plant on Yaowang Mountain was from Yangchun, Guangdong Province.

High molecular weight DNA was extracted and sequenced on the PacBio Sequel II (SMRT) and Nanopore PromethION (ONT) platforms. The former was performed at Berry Genomic Corporation Ltd. (Beijing, China) by constructing a 40 kb SMRTbell library and the latter was performed at GrandOmics Biosciences (Wuhan, China) by constructing a 1D library. For the Hi-C sequencing, DNA was manipulated after the leaves were fixed with 1% (vol/vol) formaldehyde, followed by cell lysis, chromatin digestion (DpnII), proximity-ligation treatments, and DNA recovery^52^. For NovaSeq, DNA was directly isolated using a Plant Genomic DNA Kit (DP305, Tiangen, Beijing, China) according to the manufacturer’s protocol. Both the Hi-C library and NovaSeq DNA PCR-free library were constructed and sequenced on an Illumina NovaSeq6000 system.

Cutting seedlings from patchouli plants originally collected from Yangchun (YC), Hainan (HN), Shipai (SP), Gaoyao (GY), and Indonesia (YN) were planted in our greenhouse at Guangzhou University of Chinese Medicine (Guangzhou, China). Their DNA was also extracted and sequenced on an Illumina NovaSeq6000 system.

### Genome survey

The genome size of patchouli was measured by flow cytometry and a *k*-mer analysis. For flow cytometry, according to the protocol described by Dolezel and Bartos^53^, young leaves of sequenced patchouli plants and a tomato plant used as an internal control were chopped with a sharp razor blade in OTTO I buffer (45 mM MgCl_2_·6H_2_O, 20 mM MOPS, 30 mM sodium citrate, 1% (W/V) PVP-40, 0.2% (v/v) Triton X-100, 10 mM Na_2_EDTA, 20 μL/mL β-mercaptoethanol; pH 7.5). The samples were incubated for 5 min and filtered through 70 µm Biosharp filters. The plant cell nuclei were stained by adding 1.5 mL of OTTO II buffer containing 500 µL of propidium iodide and RNAse A in the dark for 20–30 min. The relative nuclear genome size was analyzed on a BD FACSAria III flow cytometer. Each sample was repeated three times independently. Flow cytometry was used to estimate the genome size based on equal proportions of fluorescence intensities and the genome size. The average fluorescence intensity of tomato was 3932.3, and that of patchouli was 4545. The haploid tomato genome was ~900 Mb; thus, the patchouli genome size was ~1.04 Gb as 3932.3/4545 = 900/1040. For the *k*-mer analysis, Jellyfish (v2.3.0)^54^ was used to count the 19-mer frequency of genomic NovaSeq reads, and GenomeScope2.0^55^ was used to establish a detailed mathematical model of how these 19-mer frequencies could be distributed in the patchouli genome.

The chromosome number of patchouli was counted using chromosome counting technology. When the roots of the tissue-cultured seedlings grew to 1 cm, their tips were harvested at 9:00–11:00 AM. These tips were then treated with 0.0002 M 8-hydroxyl quinoline at room temperature for 3 h and subsequently fixed in Carnoy’s fixative (ethanol:acetic acid = 3:1) for at least 2 h. Then, the root tips were washed with distilled water, dissociated in 1 mol/L HCl for 2–3 min in a 60 °C water bath, crushed in improved carbol fuchsin for 20 min and treated with 45% acetic acid. The chromosomes were finally observed by microscopy.

### Genome assembly and quality control

PacBio SMRT, NovaSeq, and Hi-C reads from sequenced patchouli plants were incorporated into the genome assembly as shown in Supplementary Fig. 4. After self-correction and low-quality region trimming, SMRT long reads were mapped to a previously released patchouli chloroplast genome (NCBI accession: NC_042796.1)^56^ by Minimap2 (version 2.5-r572)^57^ with the parameters -t 96 -ax map-pb. As the mapped reads belonged to chloroplasts, the remaining unmapped reads were first assembled to obtain the nuclear genome. Canu (v2.0)^58^ was used to assemble SMRT long reads into rough contigs with the parameter genome Size = 1g. Pbmm2 and gcpp contained in smrtlink (v8.0) (https://www.pacb.com/support/software-downloads/) were used to polish these contigs by realigning the SMRT reads. The Pbmm2 alignment used the parameter -j 108 -preset SUBREAD, and gcpp polish used the parameter -j 96. Pilon (v1.22)^59^ was used for further error correction by aligning the NovaSeq reads using parameters -changes -threads 90 -minmq 10 and -fix bases, amb, gaps. The polished contigs were anchored to chromosomes with the help of Hi-C using the 3D-DNA package (v180419)^60^. The Hi-C reads were processed and aligned to the polished contigs by Juicer, and then clustered into chromosomes by 3D-DNA. The default parameters were used in all contig anchoring steps. As original anchored results always have misordered and misoriented contigs, manual correction, and validation were performed using Juicebox (v1.11.08) to obtain the final nuclear genome sequences. For the chloroplast mapped SMRT reads, Canu, Smrtlink, and Pilon were again used for assembly and polishing, and Mummer was used to analyze the completeness of the chloroplast genome. Finally, redundant sequences were manually removed to obtain the final chloroplast genome.

ONT reads and NovaSeq reads were used to assess the quality of our assembly. ONT reads >100 kb were mapped to the patchouli genome by minimap2^57^ using the parameter -t 96 -ax map-ont. A read can be defined as a mapped read only when >90% of its own length is continuously mapped to a single chromosome position. The NovaSeq reads were mapped by BWA (v0.7.16a-r1181)^61^. We searched the Arabidopsis type telomere specific motif “TTTAGGG” using seqkit (v0.13.0)^62^ and manually merged them into telomere regions according to their locations. Telomere regions located within 150 kb of the start or end of a chromosome were counted. The centromeric regions were explored based on the results of Tandem Repeat Finder (v4.09)^63^ using the recommended parameter. Two simple repeat sequences Pat_cen1 (173bp) and Pat_cen2 (174bp) were detected to define centromeric regions (Supplementary Table 15).

### Genome annotation

A combination of structure-based and homology-based approaches was employed to identify TEs and other repeat regions. First, miniature inverted repeat transposable elements (MITEs) and long terminal repeat (LTR) elements were annotated by structure-based methods. The former was performed by MITE Hunter (11-2011)^64^, and the latter was performed by LTR_retriever (v2.8.7)^65^, which integrates LTRs predicted from LTRharvest^66^ and LTR_Finder (v1.0.7)^67^. Other known repeat sequences were annotated by searching RepBase (v20170127) (http://www.girinst.org/server/RepBase/index.php) in RepeatMasker (v4.1.0) (http://repeatmasker.org). Then, RepeatMasker was used to annotate all repeat sequences by searching a library that combined MITEs, LTRs and known repeat sequences. Furthermore, RepeatModeler (v2.0) (http://www.repeatmasker.org/RepeatModeler/) was used to update the repeat sequence library by identifying the types of repeat sequences, and RepeatMasker was finally employed to mask the patchouli genome. As a byproduct of LTR_retriever, the LAI was used to assess the patchouli genome assembly quality^24^.

The repeat masked genome was used for the gene annotation. The protein-coding genes were annotated by incorporating transcriptional evidence, homology support from related species, and ab initio methods. Eighteen Patchouli RNA-seq datasets (SRR8769986, SRR7268115, SRR7268117, SRR8785265, SRR1770488, SRR7268119, SRR8756845, SRR7345998, SRR7345999, SRR7346000, SRR8755904, SRR8767850, SRR8755475, SRR8775235, SRR8775238, SRR8793583, SRR8809556, and SRR8820010) were downloaded from the NCBI SRA database, containing samples from different accessions (Yinni (YN), Shipai (SP) and Hainan (HN)), different tissues (root, stem and leaf) and different treatments (MeJA, salicylic acid (SA), abscisic acid (ABA), ethanol and light)^68–70^. After trimming by Fastp (version 0.20.1)^71^, the clean data were aligned to the patchouli genome by HISAT2 (v2.1.0)^72^, and the transcripts were assembled by StringTie (v2.1.3b)^73^ with the default parameters. The transcripts from all samples were merged and subjected to TransDecoder (https://github.com/TransDecoder/TransDecoder/wiki) in PASA (v2.4.1)^74^ for protein-coding sequence prediction and quality filtering. Only complete transcripts were retained for further analysis. The protein sequences from *Arabidopsis thaliana* (Phytozome, TAIR10), *Sesamum indicum* (NCBI, GCF_000512975.1_S_indicum_v1.0), *Solanum lycopersicum* (Phytzome, ITAG 3.2) and *Utricularia gibba* (CoGe, ID29027) were mapped to the assembled genome using Genoma (v1.6.1)^75^ to obtain high-quality protein structures. SNAP (version 2006-07-28)^76^, GeneMark-ESSuite (version 4.57)^77^, and Augustus (v3.2.2)^78^ were used for the ab initio gene prediction. They were all trained by high-quality transcripts from the last step, and then, de novo gene identification was performed according to the instruction manuals. All gene structures predicted by the above methods were integrated into a nonredundant gene set using EVidenceModeler (EVM) (v1.1.1)^74^. The weight value was set to 10 for high-quality RNA-seq transcripts, 5 for high-quality homologous proteins, and 2 for ab initio predicted transcripts. The EVM-predicted genes were further corrected with PASA (v2.4.1)^74^ to predict the untranslated regions and alternative splicings. The resulting protein models were finally functionally annotated by integrating the annotation information from InterProScan (v5.18-57.0)^79^, the NCBI nonredundant protein database (ftp://ftp.ncbi.nlm.nih.gov/blast/db/) and the eggNOG database (v5.0) (http://eggnog5.embl.de/#/app/downloads). In addition, protein-coding genes in the chloroplast were structurally and functionally annotated using the online database CpGAVAS2^80^.

Noncoding RNA was annotated using RNAmmer (v1.2)^81^ for rRNA, tRNAscan-SE (v2.0.0)^82^ for tRNA and the cmscan module in Infernal (v1.1.2)^83^ for miRNA, snRNA and snoRNA. All predictions were performed with the default parameters, with the exception of tRNAs, which were filtered with a score >40.

### Genome synteny analysis

A chromosome synteny analysis was performed using MUMmer Toolkits (ver 3.0)^84^. Nucmer was used to align each pair of chromosomes (-g 1000), delta-filter was used to filter the alignment blocks (-r -q -l 200), and show-coords was used to convert the results into a readable format. The retained blocks were dot plots, which were visualized by in-house scripts.

Syntenic genes between subgenome homoeologous chromosomes and tandemly duplicated genes in each chromosome were extracted from the gene synteny analysis results by MCScanX^85^ using the default parameters. The longest proteins encoded by each gene in subgenomes A and B were selected and blasted against each other using Blastp (v2.6.0). The *E*-value was set to 1E-5, and the coverage percentage was set to 50% to obtain an accurate homologous gene pair alignment.

### Structural variation detection

The structural variations between inter- and intrasubgenome homeologous chromosomes were detected by SYRI (v1.4)^86^ using alignment blocks produced by MUMmer (ver 3.0)^84^. In contrast to the genome synteny analysis, more accurate parameters were used in the genome comparison by nucmer (-maxmatch -l 40 -g 90 -b 100 -c 200) and the syntenic block filtration by delta-filter (-m -i 90 -l 100). SYRI was performed with the default parameters.

### Subgenome enrichment for k-mer identification

The subgenome-enriched *k*-mer sequence was identified using the modified method described by Session et al.^87^. Briefly, short 13-mer sequences in the whole patchouli genome were counted and filtered when the occurrence count was <1000. Then, the occurrence frequency of the retained 13-mers was counted in each chromosome. A 13-mer was defined as being enriched when it showed at least twofold occurrence in all 31 chromosomes from the A or B subgenome relative to its homoeologous chromosome from the B or A subgenome, respectively. Jellyfish (v2.3.0)^54^ was used to count 13-mers, and pheatmap^88^ in R was used to hierarchically cluster the enriched 13-mers into two groups with highly correlated distributions.

### Phylogenetic analysis and divergence time estimation

In total, 11 species were used for the phylogenetic tree construction, including patchouli. Their respective protein sequences were collected. In addition to the four species described in the “genome annotation” section, the sequences of *Oryza sativa* (GCF_001433935.1_IRGSP-1.0), *Olea europaea* (742605.1_O_europaea_v1), and *Milulus guttatus* (GCF_000504015.1_Mimgu1_0) were downloaded from the NCBI database; those of *Scutellaria barbata* (GWHAOTP00000000) and *Scutellaria baicalensis* (GWHAOTC00000000) were downloaded from Genome Warehouse; and those of *Salvia miltiorrhiza* were downloaded from ftp://danshen.ndctcm.org:10402. The patchouli chromosomes were divided into four mini-subgenomes I_A_, II_A_, I_B_, and II_B_. To remove redundancies caused by alternative splicing, only gene models that encoded the longest protein sequence were retained for each gene locus. All filtered protein sequences of these plants were compared with each other using Blastp (*E*-value < 1E-10) (v2.6.0)^89^ and clustered into orthologous groups using OrthoMCL (v2.0.9)^90^. A single-copy orthologous group containing 587 genes from ten species and four patchouli mini-subgenomes was selected.

First, 587 gene sequences from each species were concatenated into 14 superprotein sequences. Multiple protein sequence alignments were generated by MAFFT (v7.471)^91^ and then converted into the PHYLIP format. Then, a maximum-likelihood tree was constructed with RAxML (v8.0.19)^92^ while 100 bootstrap replicates were performed. Second, to consider the effect of incongruence among the gene trees on the species tree topology, we used a coalescent-based species tree approach^93^. Phylogenetic analyses of individual gene alignments were also conducted in RAxML, and the resulting 587 gene trees were collapsed with bootstrap support <10% and then used as input for species tree inference with ASTRAL-III^94^ using multi-locus bootstrapping with 100 replicates. The discordance among the gene trees and the ASTRAL-III species tree was assessed using Phyparts^95^ and visualized using phypiecharts.py (https://github.com/mossmatters/MJPythonNotebooks/blob/master/phypartspiecharts.py).

The divergence times were estimated under a relaxed clock model using the Mcmctree program in PAML (ver4.9e)^96^. The “JC69” model in Mcmctree was run as sample number set to 10,000, sample frequency set to 1000 and burn-in iteration set to 5000. The calibration points were obtained from the TimeTree database (http://www.timetree.org/).

### Analysis of chromosome doubling events

Paralogous or orthologous gene pairs between any combination of patchouli subgenome A, patchouli subgenome B, the *Scutellaria barbata* genome and the *Vitis vinifera* genome were identified by the pipeline used for the patchouli gene syntenic analysis. The Ks of the gene pairs was calculated using the Python script synonymous_calc.py (https://github.com/tanghaibao/bio-pipeline/tree/master/synonymous_calculation). The 4DTv of the gene pairs was calculated using the Perl script Calculate_4DTV_correction.pl^97^.

Three rounds of Ks corrections were performed^98^. First, as patchouli and *Scutellaria barbata* had the same divergence time from *Vitis vinifera*, the Ks peaks shared between *Vitis vinifera* and patchouli subgenome A, *Vitis vinifera* and patchouli subgenome B, *Vitis vinifera* and *Scutellaria barbata* were calibrated using the Ks peak between *Vitis vinifera* and *Scutellaria barbata* as a reference. Second, as Lamiaceae underwent a WGD event^99^, the Ks peaks shared within patchouli subgenome A, patchouli subgenome B, and *Scutellaria barbata* were calibrated using the Ks peak within *Scutellaria barbata* as a reference. Third, as patchouli subgenome A and patchouli subgenome B evolved from the same progenitor, the peaks shared within patchouli subgenome A and B were calibrated using the Ks peak within patchouli subgenome A as a reference.

### TE classification and comparisons

To analyze the TEs more precisely, a repeat sequence library constructed during the repeat annotation was processed with the Tephra toolkit (version 0.13.1) (https://github.com/sestaton/tephra). The identification modules (findltrs, sololtr, findtrims, findnonltrs, findtirs, and findhelitrons), classification modules (classifyltrs and classifytirs) and insertion time estimation module (age) were used. In brief, full-length LTR-RTs, non LTR-RTs, TIR transposons, and helitrons were refined by analyzing the structure. Full-length LTR-RTs were first classified into Gypsy, Copia and Unclassified superfamilies based on the presence and order of canonical protein-coding domains in these taxonomic groups. The TIR elements were classified into superfamilies based on the characteristic structural features shared across eukaryotes.

All TEs were grouped into subfamilies based on the shared similarity between their sequences by applying the Tephra “classifyltrs” modules. The LTR region and coding/binding domains of the LTR-RT elements were used for the subfamily classification. The insertion times of LTR-RTs and TIRs were estimated based on the divergence between their LTR sequences and TIR sequences using the K80 substitution model from PAML (ver4.9e)^96^.

The phylogenetic trees of the LTR-RT superfamilies were specifically constructed. Full-length LTR elements were subsequently translated into amino acid sequences in two directions (forward and reverse) and three frames (0, 1, 2). The translated sequences were mapped against the Pfam database (ftp://ftp.ebi.ac.uk/pub/databases/Pfam)^100^ using InterProScan (v5.18-57.0)^79^ to search for protein domains with *E*-values < 1E-5. The PF00078 domain was selected for Gypsy, the PF07727 domain was selected for Copia, and the PF03732 domain was selected for Unclassified LTR-RTs. The sequences of these domains of each superfamily were extracted and aligned using MAFFT (v7.471)^91^. The phylogenetic trees of the three LTR superfamilies were finally constructed using FastTree (v2)^101^.

The elements in the LTR-RT Gypsy and Copia superfamilies were aligned to the Viridiplantae_v3.0 database (http://repeatexplorer.org/?page_id=918)^102^ using Blastp (v2.6.0)^89^ with the parameters of *E*-values < 1E-5 and max_target_seqs = 1. They were classified into several lineages according to the annotation of their blasted elements in the database and finally modified based on the structures of the phylogenetic trees. If >90% of the elements in a branch belonged to the same lineage, all elements in this branch were named after this lineage. The elements in the Unclassified LTR-RT superfamily were classified directly according to the structure of its phylogenetic tree.

### Resequencing analysis

The SLAF-seq reads of 22 patchouli accessions were downloaded from the NCBI SRA database (SRP057143, https://www.ncbi.nlm.nih.gov/sra/?term=SRP057143)^21^. In total, 15,457,835 reads were produced and 61,334 SLAF (specific-locus amplified fragments) were obtained in this dataset. For each patchouli accession, the most one sequenced 1,065,230 reads and obtained 52,039 SLAF, the least one sequenced 400,385 reads and obtained 38,603 SLAF. Reads from each accession were mapped to the patchouli genome using the mem alignment mode in BWA (v0.7.16a-r1181)^61^ and then filtered based on a map quality > 30. An in-house script was used to count the read number of each chromosome.

For the whole genome resequencing datasets produced by ourself, including YC, HN, YN, SP, GY and the sequenced patchouli, the reads were mapped to the genome by BWA after trimming by Fastp (version 0.20.1)^71^. GATK (version 4.1.8.1)^103^ was used to call and extract SNPs following the instruction manual. The *π*-value of each SNP was calculated by VCFtools (v0.1.16)^104^ and the genetic diversity in specific regions (genes or chromosomes) was calculated as follows: sum of all SNP *π*-values in this region divided by the total length of this region.

### Gene expression and functional analysis

Whole genome-wide gene expression was estimated in the 18 RNA-seq datasets used in the gene annotation. The sequenced reads were mapped to the genome by HISAT2 after trimming by Fastp (version 0.20.1)^71^. Gene expression was estimated using StringTie (v2.1.3b)^73^ when a gene annotation structure was provided with the -G option. The expression of protein-coding genes was normalized using the number of reads per kilobase of exon sequence in a gene per million mapped reads (FPKM). A KEGG pathway analysis was performed using KOBAS 3.0^105^, and only pathways containing more than five target genes with enrichment *q*-values < 0.05 were considered significantly enriched.

### Identification of genes in the terpene biosynthesis pathway

Enzyme-coding genes in the MVA and MEP pathways of terpene biosynthesis were identified by an incorporated approach. The Pfam domains of genes encoding enzymes in each catalytic step were first searched in the literature, and then, patchouli genes containing these domains were selected as candidates. They were further confirmed by a conserved domain analysis when aligned with validated genes from other species and the phylogenetic tree construction. Multiple sequences were aligned using MUSCLE (v3.8.31), and a maximum-likelihood tree was constructed with MegaX (version 10.0.5)^106^.

### Biochemical assay of *Pat_49G072100*

The cloning, recombinant protein purification and enzyme assay of *Pat_49G072100* were conducted following previously reported pipelines^107^. In brief, the total RNA was extracted, and cDNA was synthesized from mature leaves of sequenced patchouli plant. The primers used for *Pat_49G072100* cloning were 28a-PTS1-F (ATGGAGTTGTATGCCCAAAGTGT) and 28a-PTS1-R (AATATGGAACAGGGTGAAGGTACAA). The pET-28a plasmid was used for protein expression, and the CDS of *Pat_49G072100* was inserted between the BamHI and XhoI restriction enzyme cutting sites. The recombinant protein was induced by 0.5 mM IPTG at 220 r/min and 16 °C overnight, and then purified using a Ni-NTA Superflow Column (QIAGEN, Germany). Approximately 50 μg purified protein were incubated with 5 μg FPP in buffer solution (50 mM HEPES, 5 mM MgCl_2_, 5 mM DTT, pH 7.5) at 30 °C for 2 h. The reaction mixture was then extracted with 200 μL hexane, by vortexing 5 min, standing 5 min, and centrifuging at 13,201 × *g* for 5 min to separate the organic phase. The extracted compounds were analyzed using GC-MS.

### Determination of patchouli alcohol in different accessions

Approximately 300 mg of mature leaves from the patchouli SP, YN, and HN accessions were harvested and immediately frozen in liquid nitrogen. The leaves were ground into powder, and half of the powder was weighed and processed^21^. The extracts were filtered through a 0.22 μm microfilter prior to the GC-MS analysis. The identification of patchouli alcohol was achieved by comparison to the authentic standard (A0300, Chengdu Must Bio-Technology, Chengdu, China). Standard curves of serial dilutions of authentic standards were used for the quantification. The patchouli alcohol content in each sample was further converted relative to the amount of tissue utilized. The measurements were performed with three biological replicates of each accession.

### GC-MS analysis

The GC-MS analysis was performed on a 7890A/5975C GC-MS unit (Agilent Technologies, Santa Clara, CA, USA) equipped with an HP-Intiowax polyethylene glycol column. The injector temperature was 250 °C. Helium was used as the carrier gas at a constant flow rate of 1.0 ml/min. For the patchouli alcohol determination in different accessions, 1 μl of sample was injected in the split mode (20:1) onto the column. The temperature gradient was started at 60 °C for 2 min, increased to 250 °C at a rate of 6 °C/min, and then held for 3 min. For the enzyme assay of *Pat_49G072100*, the sample was injected in the splitless mode, and the temperature gradient was started at 50 °C for 1 min, increased to 100 °C at a rate of 10 °C/min, and then held for 2 min. The ion source of the mass spectrometer was set at 230 °C, and the transfer line temperature was 300 °C.

### Real-time RT-PCR

In addition to the metabolite detection, the remaining mature leaf samples were used for RNA extraction. An RNAprep Pure Plant Kit (DP432, TIANGEN, Beijing, China) was used according to the manufacturer’s instructions. After treatment with DNase I, 5 µg of RNA were used for cDNA synthesis using an Evo M-MLV RT Kit with gDNA Clean for qPCR II Kit (AG11711, Accurate Biology, Changsha, China) according to the manufacturer’s instructions. Real-time PCR was performed on a Roche LightCycler 480 II system with a SYBR Green Premix Pro Taq HS qPCR Kit (AG11701, Accurate Biology, Changsha, China). The primers used for *PatPTS* were PatPTS-F (TGGGTGCTGCCTCTCGTCCTC) and PatPTS-R (TGCGTTGTGGACTTGATTCG); for the internal reference *Pat18S*, the primers were Pat18S-S (TCGCCGTTCGGACCAAATAA) and Pat18S-AS (CGATGGTTCACGGGATTCTGC). The relative expression level was calculated using the 2^−△△CT^ method.

### Reporting summary

Further information on research design is available in the Nature Research Reporting Summary linked to this article.

## Supplementary information

Supplementary Information

Description of Additional Supplementary Files

Supplementary Data 1

Supplementary Data 2

Supplementary Data 3

Reporting Summary

## Data Availability

All sequencing data used in the genome assembly and information concerning the assembled genome Patchouli_v1.0 have been deposited in the Genome Sequence Archive (GSA) database under Accession Number CRA004172 and the Genome Warehouse (GWH) database under Accession Number GWHBAZF00000000 in the BIG Data Center. Source data are provided with this paper.

## Source data

Source Data
