# Supplementary material for: Chromosome-level and haplotype-resolved genome provides insight into the tetraploid hybrid origin of patchouli
Source: Nat Commun. 2022 Jun 18;13:3511. doi: 10.1038/s41467-022-31121-w (PMC9206139; doi:10.1038/s41467-022-31121-w)
Supplement: Supplementary file 1 — Supplementary Information [file 41467_2022_31121_MOESM1_ESM.pdf]

Chromosome-level and haplotype-resolved genome provides insight into the tetraploid hybrid origin of patchouli

Shen *et al.*

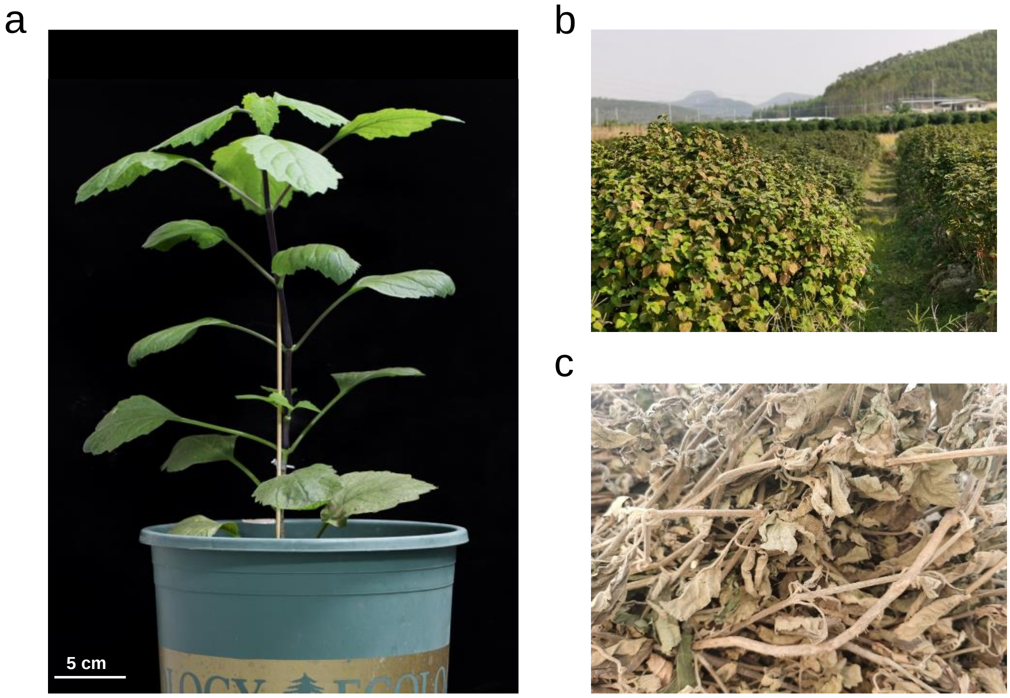

Supplementary Fig. 1. Morphology of patchouli. a, Tissue culture seedling of the patchouli plant we sequenced. **b,** The mature patchouli plants in field. **c,** The dried aerial parts of patchouli used in the pharmaceutical and perfumery industries**.**

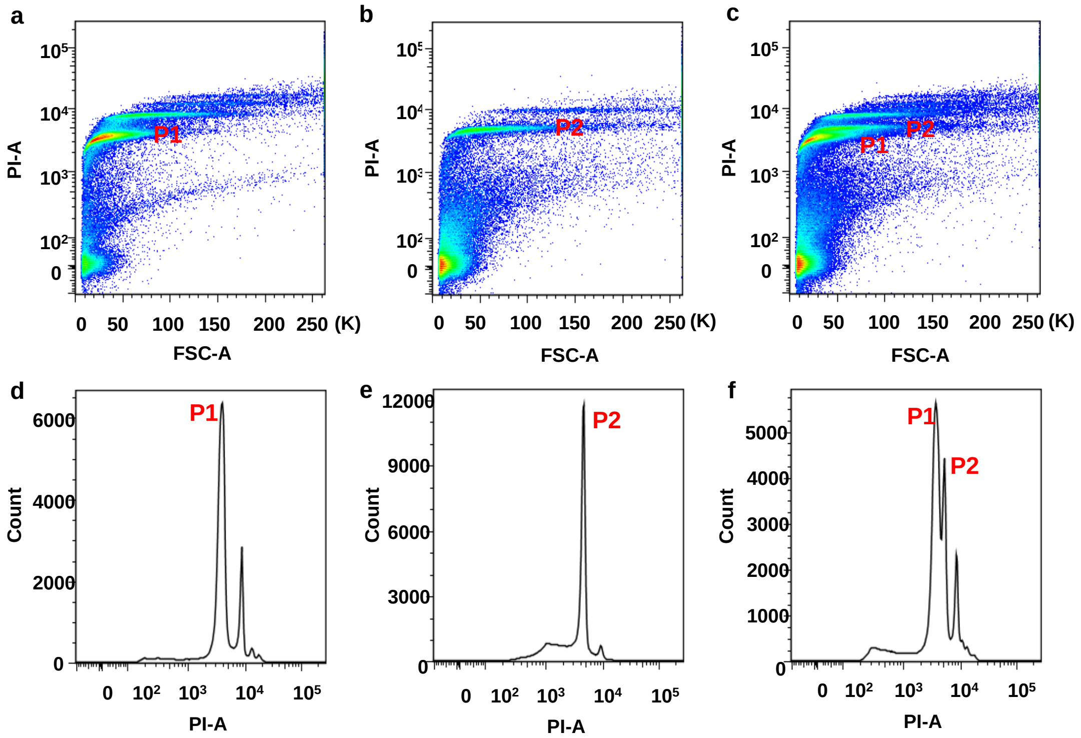

Supplementary Fig. 2. Estimation of patchouli genome size by flow cytometry using tomato as an internal standard. a-c, Flow cytometry signals for tomato leaf cells (a), patchouli leaf cells (b) and their mixture (c). **d-f,** Histograms of relative fluorescence intensities for tomato leaf cells (d), patchouli leaf cells (e) and their mixture (f).

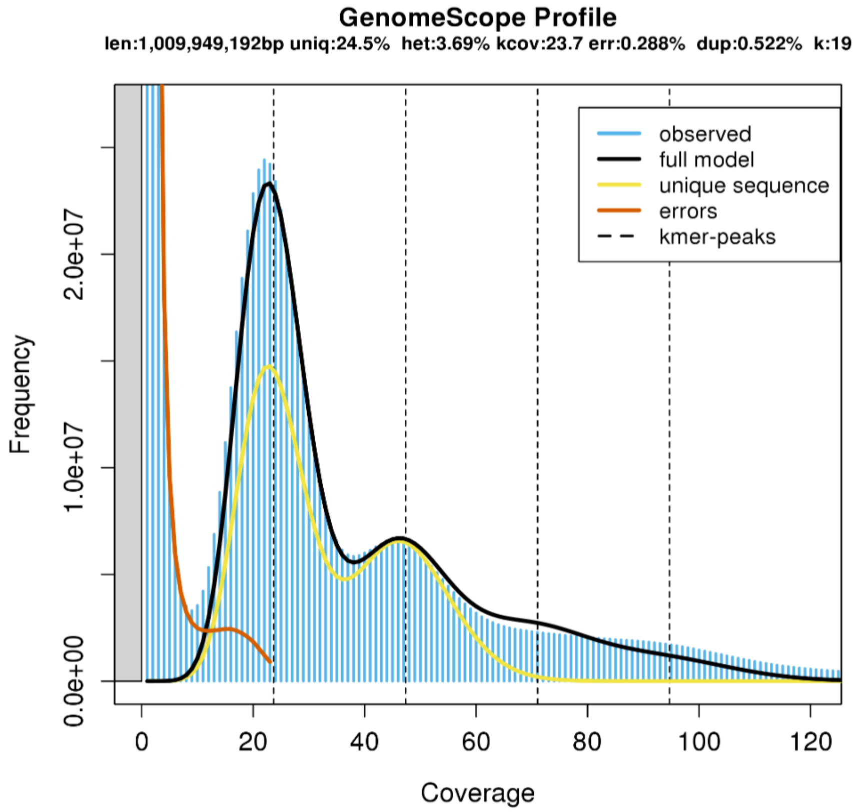

Supplementary Fig. 3. 19-mer survey result for the patchouli genome. Approximately 60× NovaSeq reads were used in this genome survey.

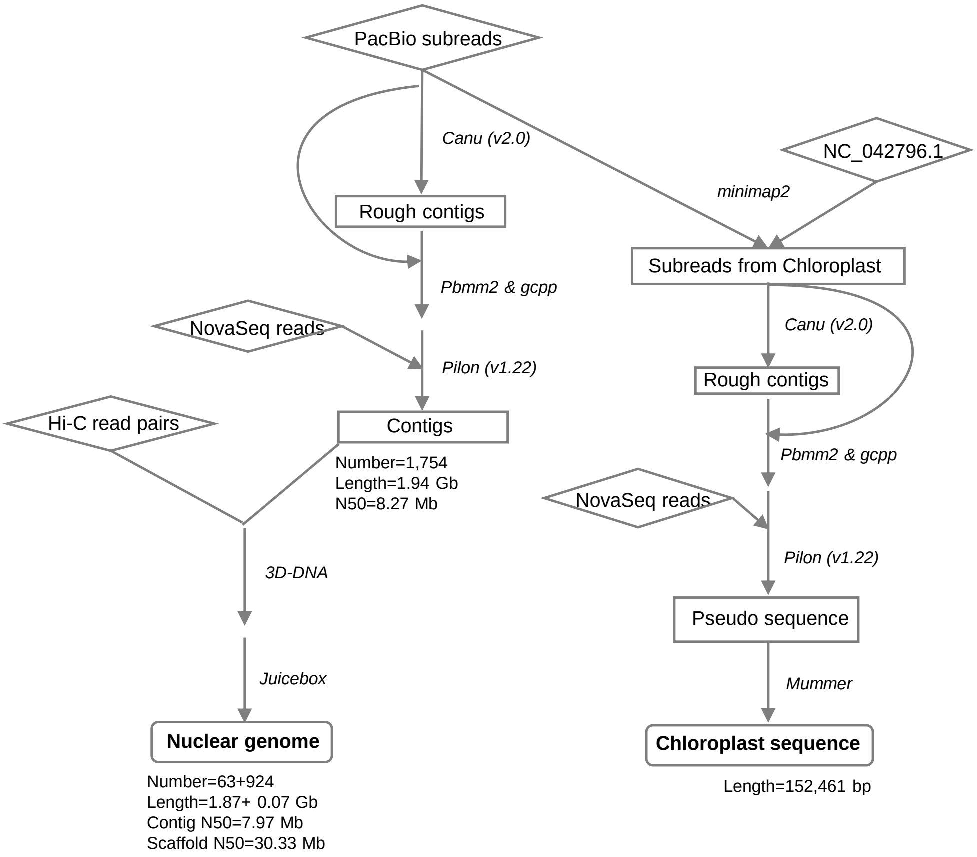

Supplementary Fig. 4. Strategies and tools used for patchouli genome (nuclear and chloroplast) assembly in this project. Rhomboids indicate the input dataset, rectangles indicate intermediate processes, and italic letters indicate the software we used.

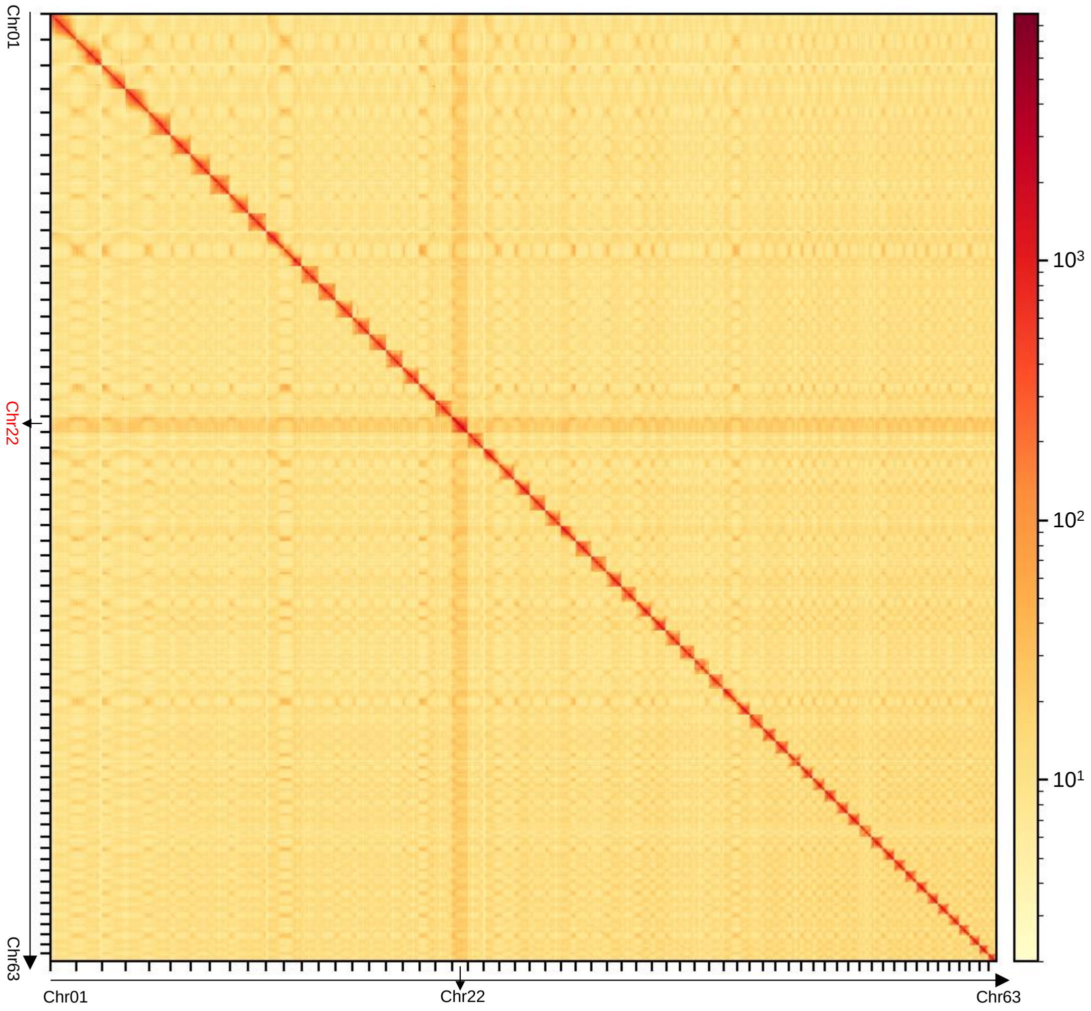

Supplementary Fig. 5. Overview of the Hi-C count matrix for assembled chromosomes. The degree of interaction between 500 kb windows of the patchouli genome is shown by colorful dots. The redder dot, the denser interaction.

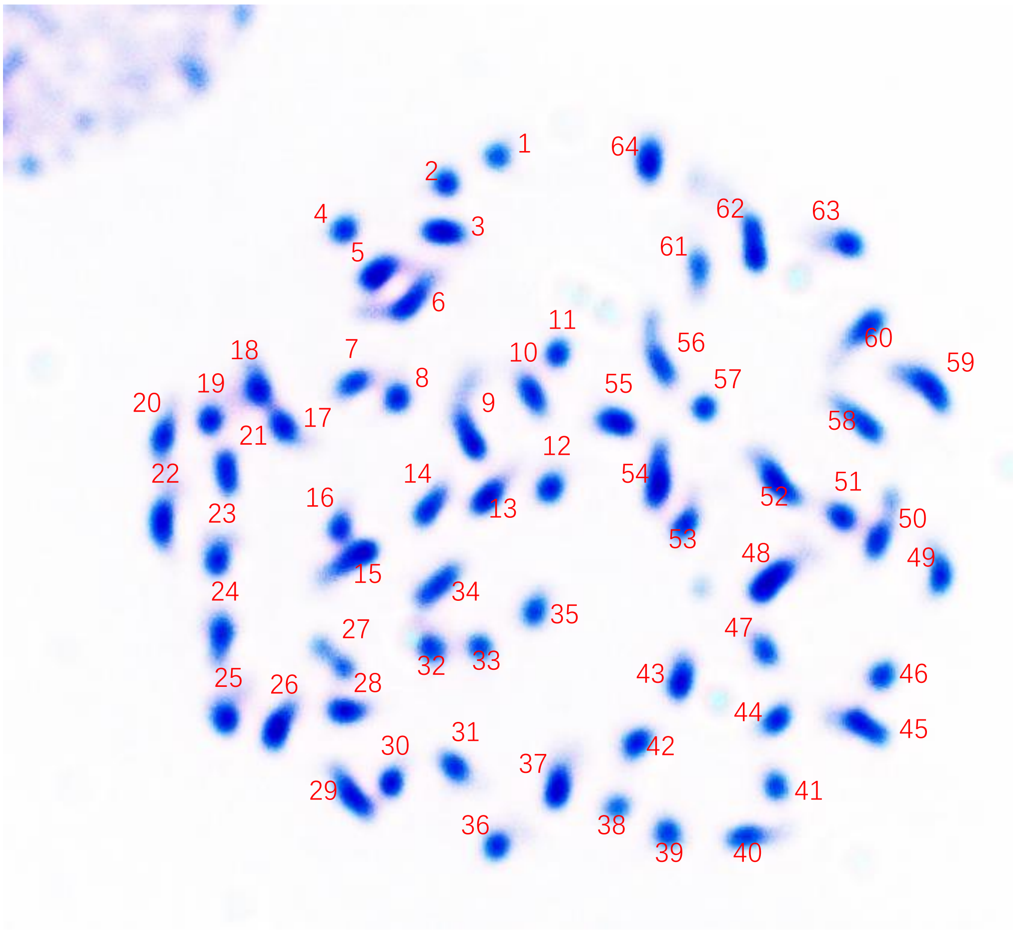

Supplementary Fig. 6. Chromosome counting for patchouli root tip cell.

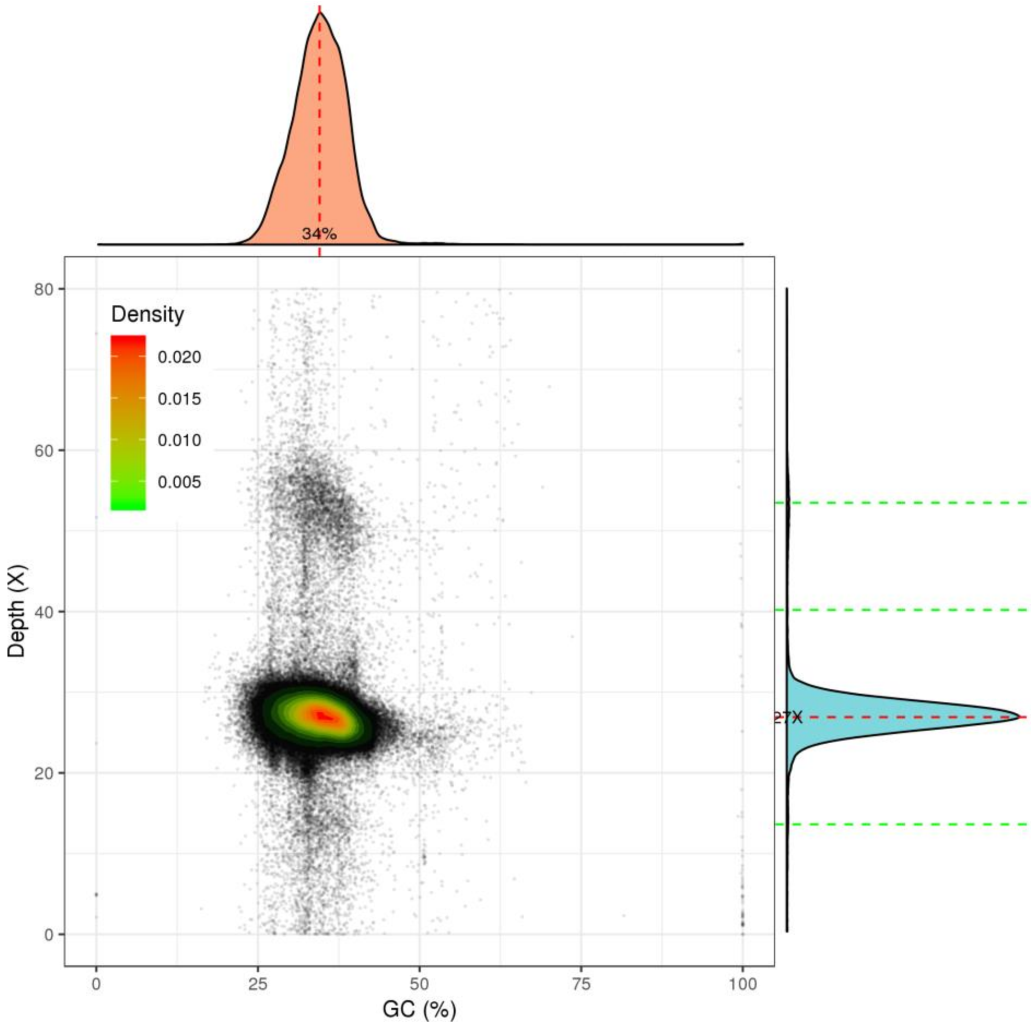

Supplementary Fig. 7. Dot plot and density distribution for GC content and read depth of remapped NovaSeq reads in assembled patchouli genome. Sequence in continuous 10 kb windows were counted.

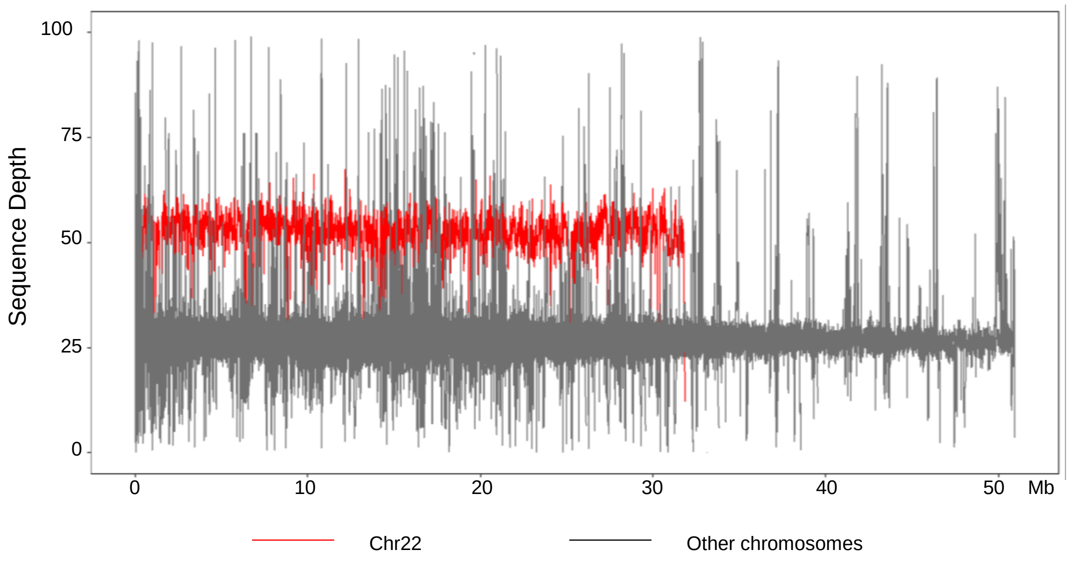

Supplementary Fig. 8. Remapped NovaSeq read depth distribution for continuous 10 kb windows of each chromosome in the assembled patchouli genome.

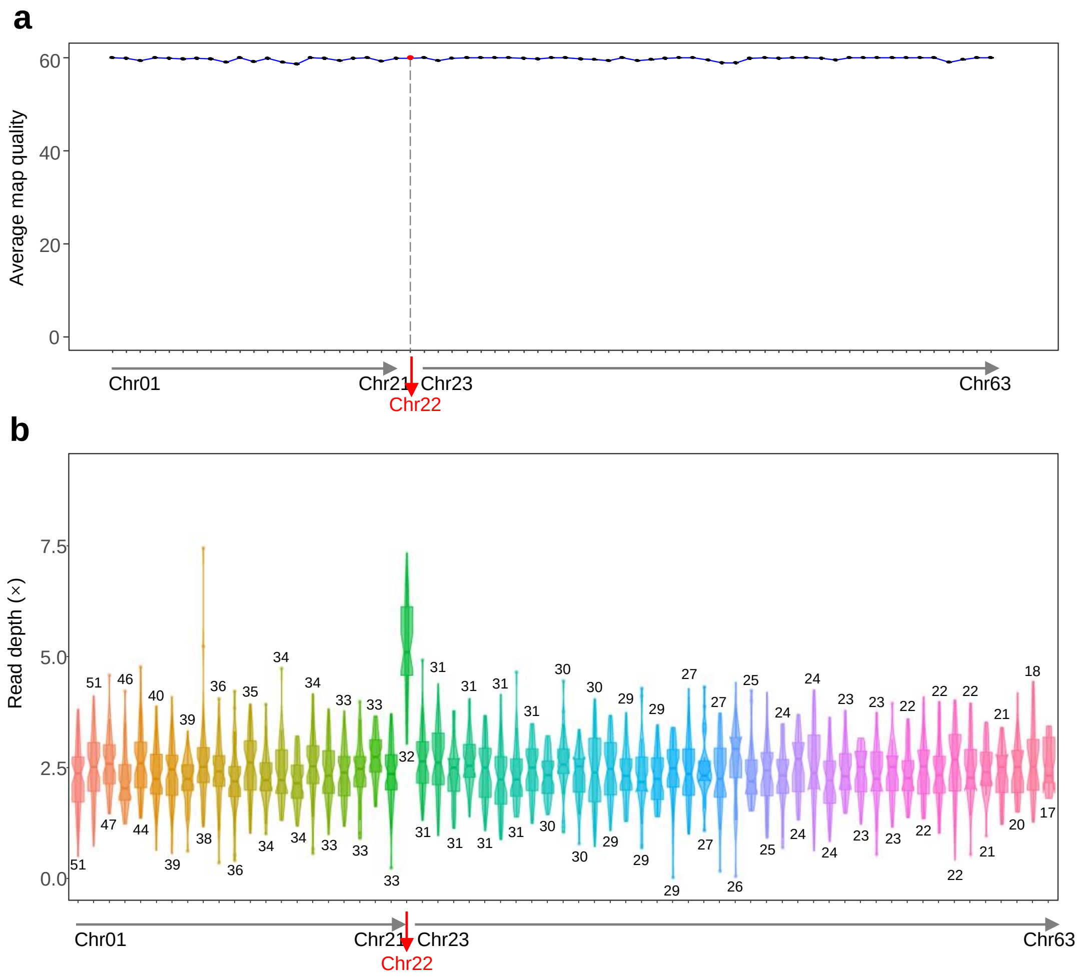

Supplementary Fig. 9. Mapping statistics of ultralong ONT reads. a, Average read map quality of each chromosome in the assembled patchouli genome. **b,** Violin plot and box plot of read depth distribution for continuous 1 Mb windows of each chromosome in the assembled patchouli genome. The number of window used for each boxplot was labeled. Boxplot shows the median and 25-75% range, 1.5× interquartile range and outliers. Source data are provided as a Source Data file.

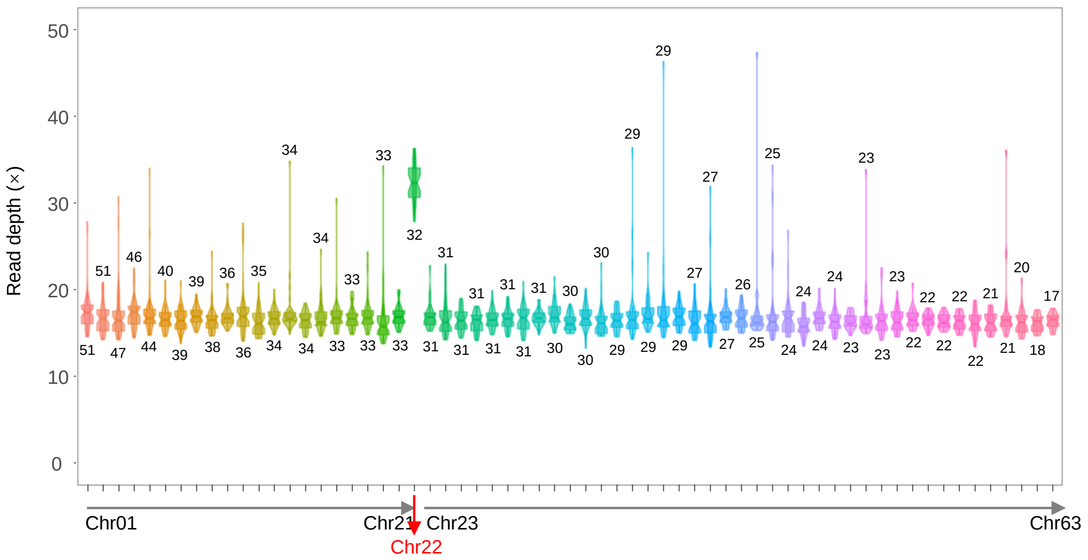

Supplementary Fig. 10. Mapping statistic for NovaSeq reads of the patchouli plant from Yangchun. Violin plot and box plot of read depth distribution for continuous 1 Mb windows were shown. Boxplot shows the median and 25-75% range, 1.5× interquartile range and outliers. The number of window used for each boxplot was labeled. Source data are provided as a Source Data file.

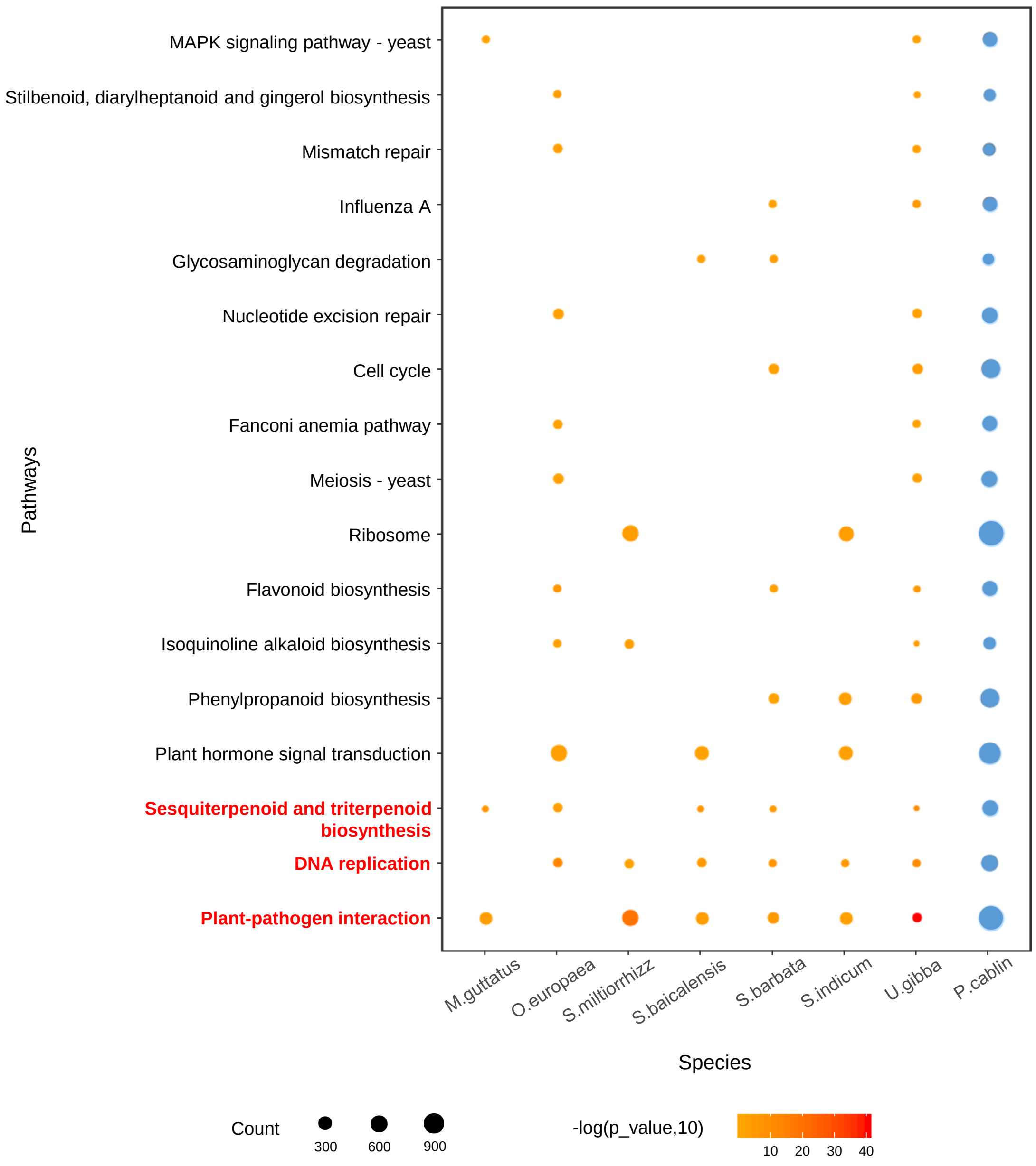

Supplementary Fig. 11. Pathways enriched in patchouli when compared with other species of Lamiales. A pathway was considered enriched in patchouli, when Fisher's exact test yielded p <0.01 when compared with other species. Only pathways enriched at least two times are shown. In Fisher's exact test, the background for each species was the number of annotated genes for this species. Blue dots for patchouli represent only the gene count. Fisher's exact test were performed using 95 percent confidence interval and two-sided alternative hypothesis.

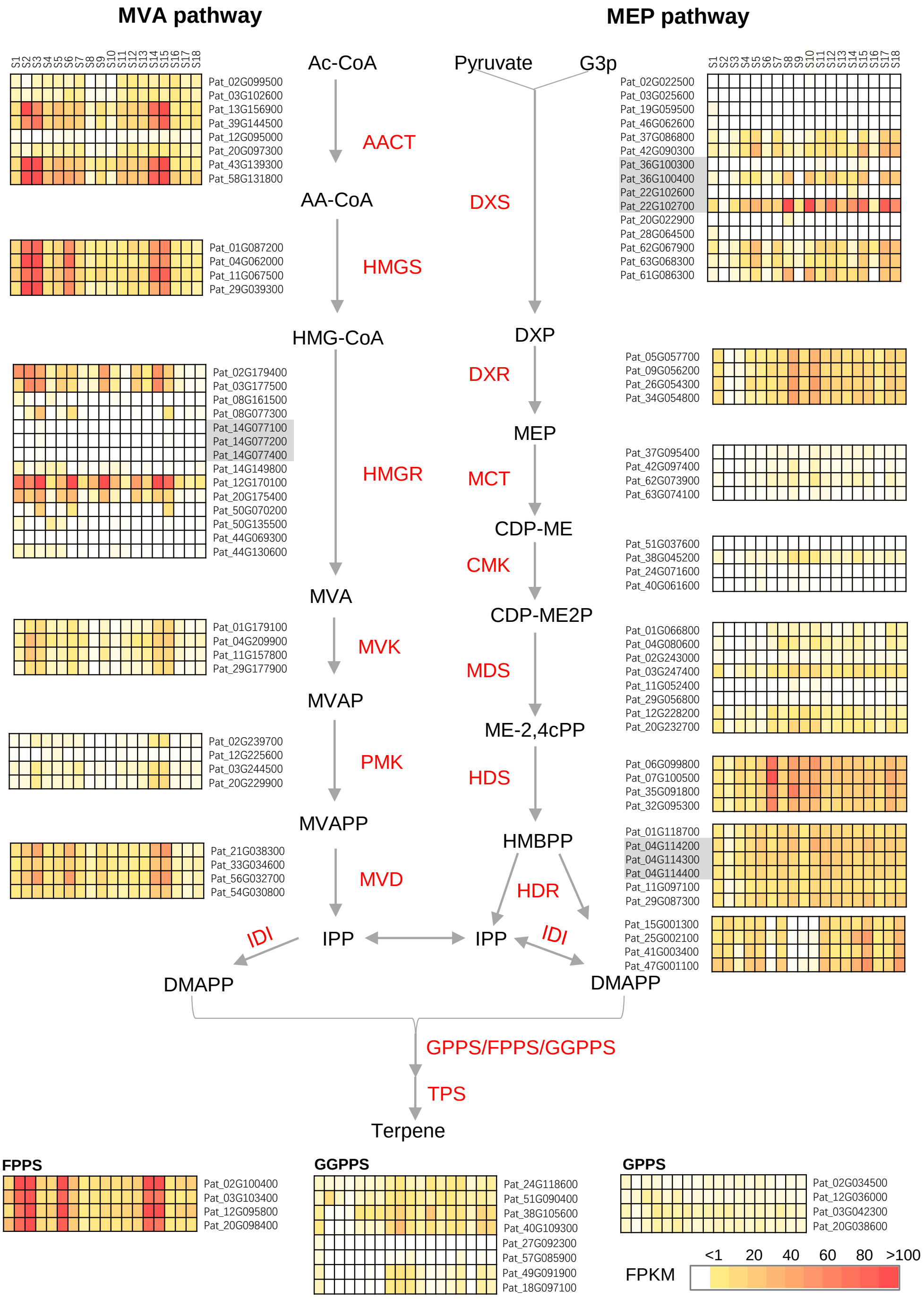

Supplementary Fig. 12. Patchouli genes participating in terpene biosynthesis and their expression patterns in different RNA-seq datasets. Genes were first blasted against their homologous genes in other species and then filtered by phylogenetic position among homologous genes and functional protein domains. NCBI accession numbers for RNA-seq datasets: S1 (SRR8769986), S2 (SRR7268115), S3 (SRR7268117), S4 (SRR8785265), S5 (SRR1770488), S6 (SRR7268119), S7 (SRR8756845), S8 (SRR7345998), S9 (SRR7345999), S10 (SRR7346000), S11 (SRR8755904), S12 (SRR8767850), S13 (SRR8755475), S14 (SRR8775235), S15 (SRR8775238), S16 (SRR8793583), S17 (SRR8809556), and S18 (SRR8820010).

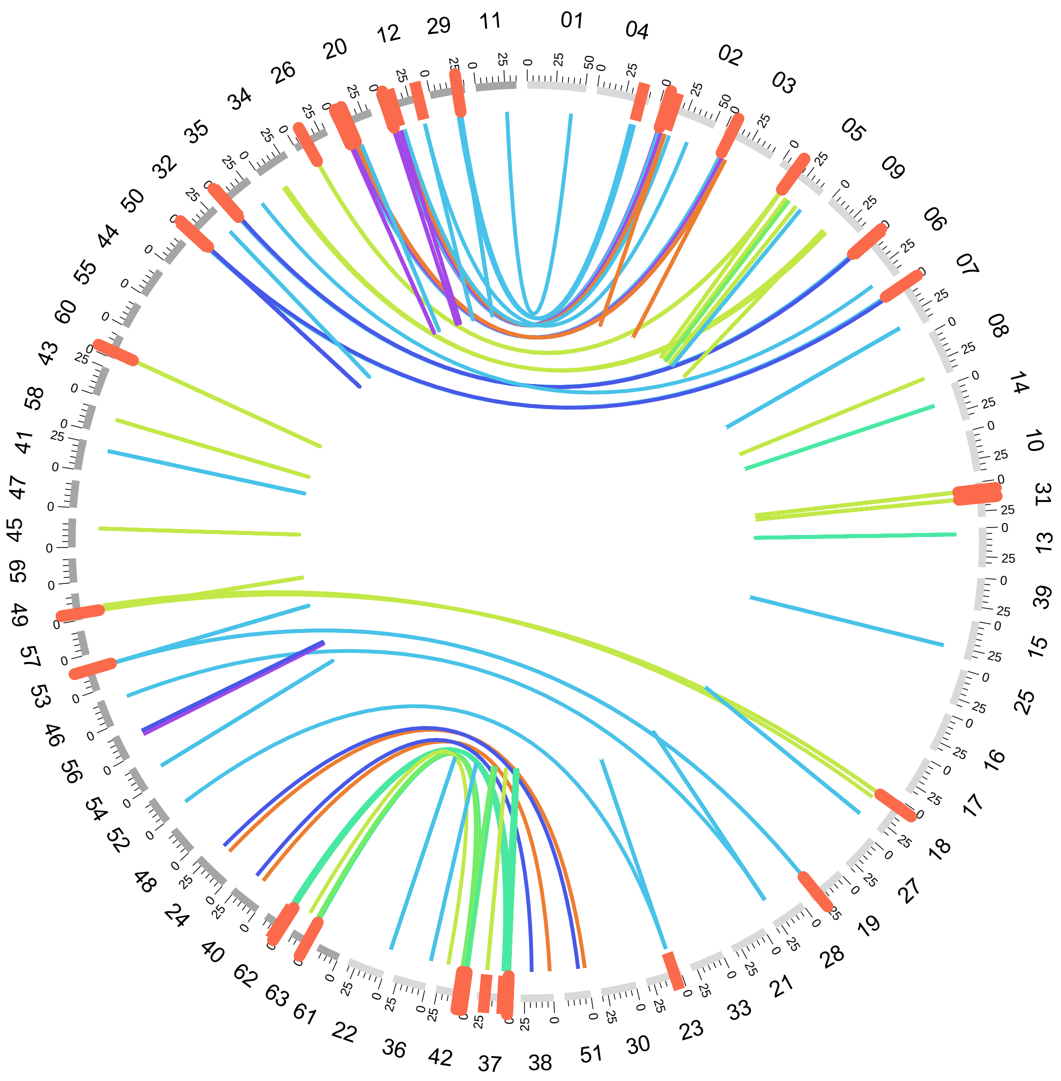

Supplementary Fig. 13. Circos distribution of 266 TPS genes. Tandemly duplicated genes are tiled as orange blocks. Genes in intersubgenome syntenic gene pairs are linked together, and nonsyntenic genes are linked to themselves. The color of the linking line is the same as the family color shown in Fig. 2a. The numbers in the outermost circle are chromosome IDs.

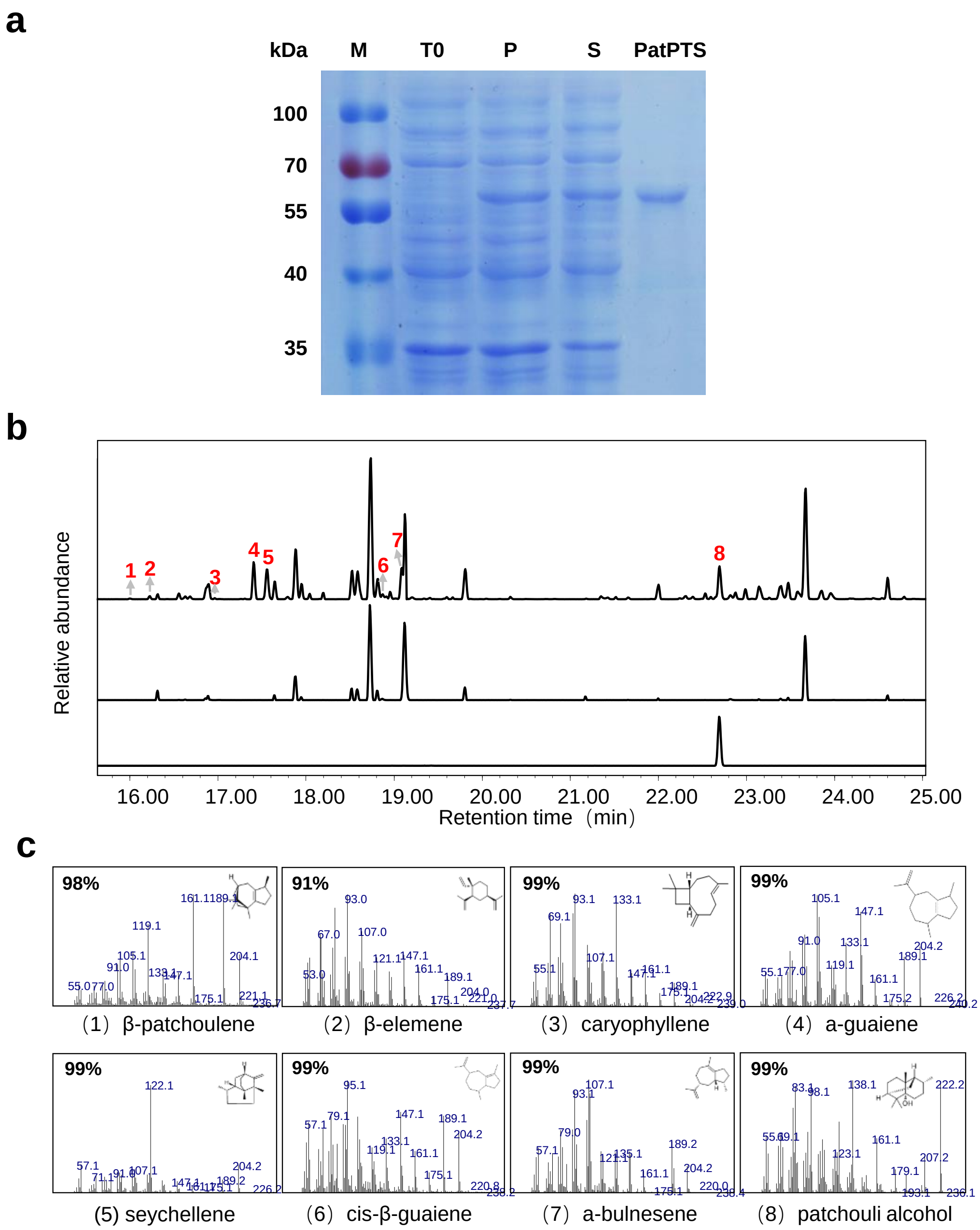

Supplementary Fig. 14. Biochemical assay of PatPTS. a, SDS-PAGE analysis of affinity-purified recombinant PatPTS expressed in E. coli. Lane M, molecular weight standard; lane T0, total protein before IPTG induction; lane P, pellet protein fraction; lane S, soluble protein fraction; lane PatPTS, eluent recombinant PatPTS protein. The gel was stained for total protein using coomassie blue. The experiment was repeated 3 times independently. **b,** GC-MS analysis of the sesquiterpene products generated by the recombinant PatPTS (up panel), blank control of pure recombinant PatPTS protein (medium panel) and standard substance of patchouli alcohol (bottom panel). Products were identified and marked by numbers in the chromatogram. **c,** Mass spectra, common names and library similarity of the identified sesquiterpenes.

TE	Class	Order	Superfamily	Number of total elements	Number of multiple family	Number of element in multiple family	Number of singleton family
TE	Class I	LTR	Copia	6,271	637	3,611	2,660
			Gypsy	5,396	425	2,603	2,793
			Unclassified	4,204	240	1,717	2,487
		Non-LTR		314	23	46	268
	Class II	TIR	Tcl-mariner	4,877	167	1,154	3,723
			hAT	168	13	40	128
			Mutator	686	68	391	295
			CACTA	177	9	35	142
			Unclassified	28,336	281	2,427	25,909
		Helitron		2,948	543	1,086	1,862

Supplementary Fig. 15. Statistics for full-length TEs and the sub-families to which they belong. Sub-families containing only one element were defined as singleton families, and families containing more than one element were defined as multiple families.

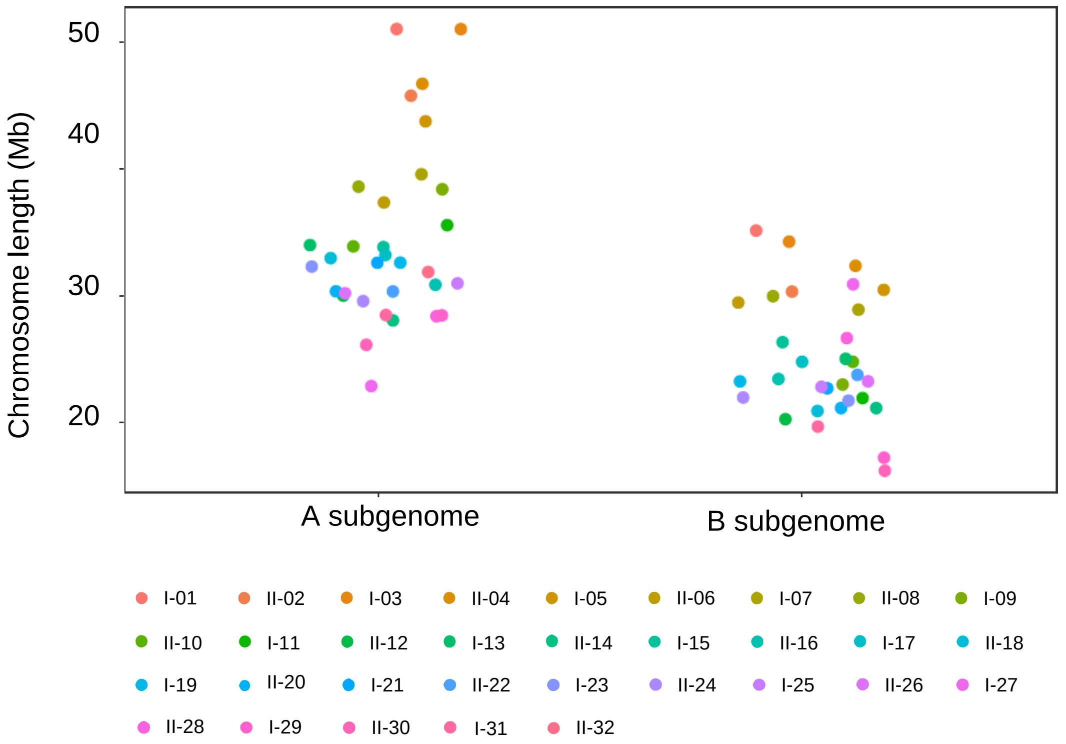

Supplementary Fig. 16. Chromosome length comparison between subgenomes A and B. Source data are provided as a Source Data file.

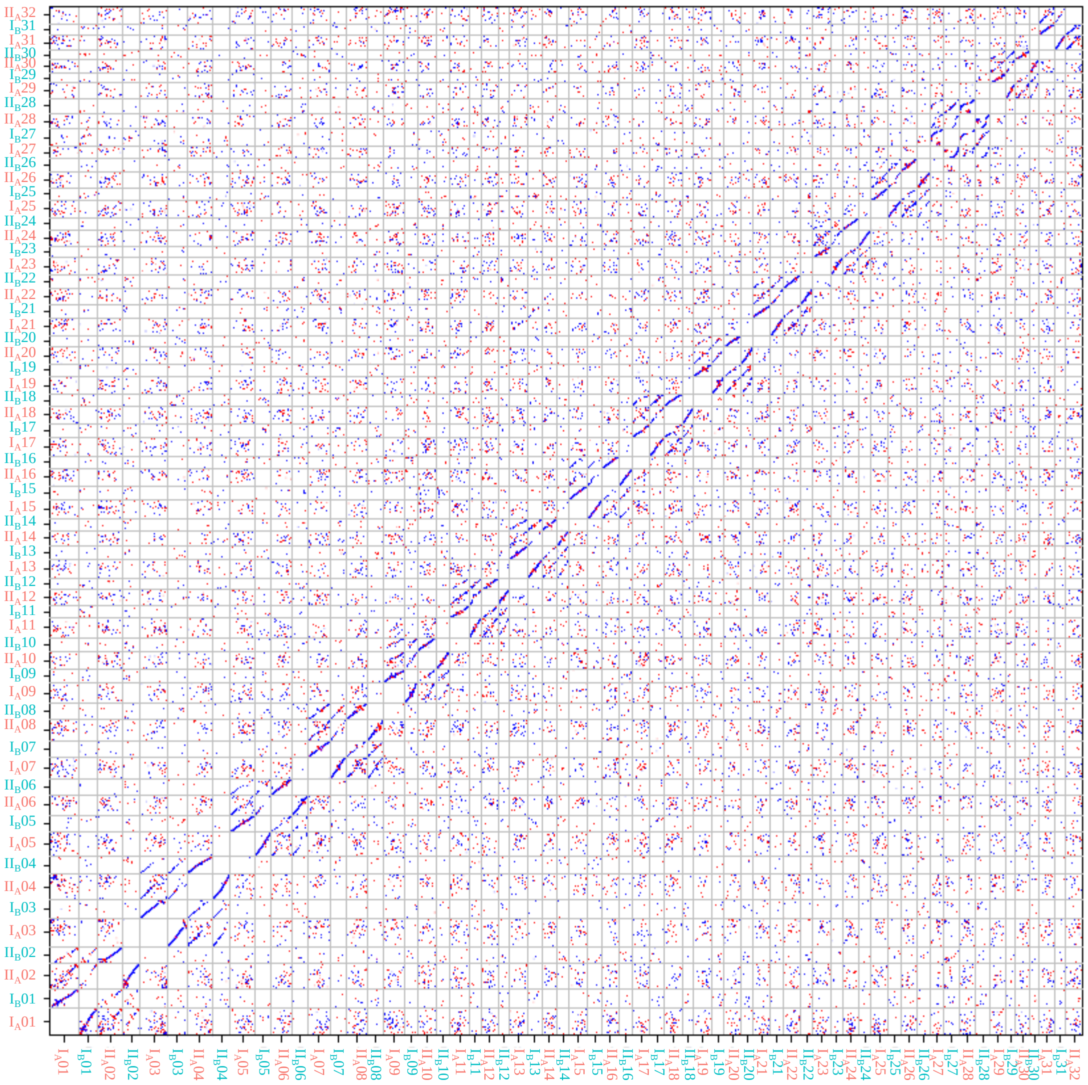

Supplementary Fig. 17. Dot plot visualization of collinearity between 63 chromosomes. Only collinearity regions larger than 5 kb are shown. Blue dots represent collinearity in the same direction, while red dots represent collinearity in the opposite direction.

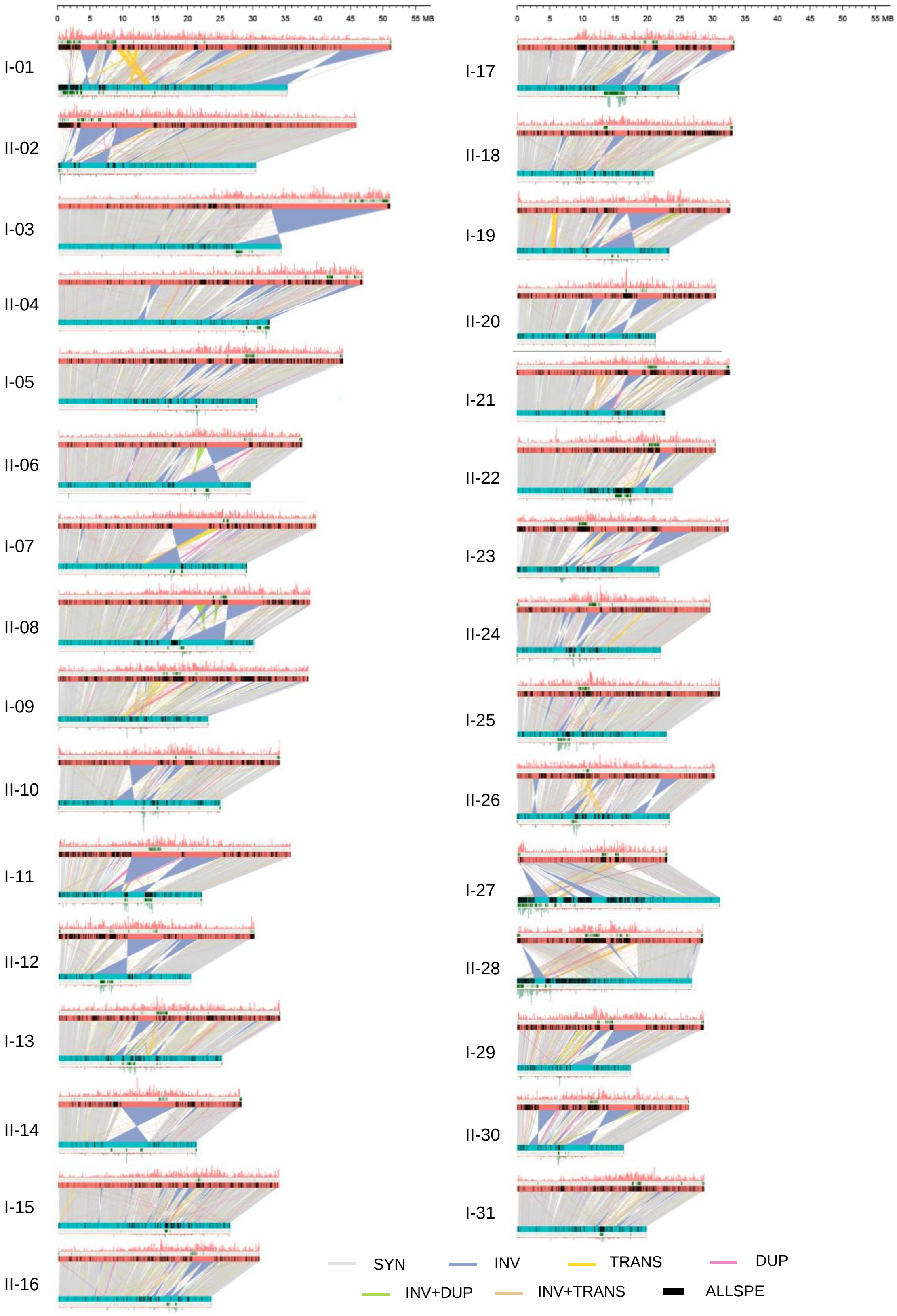

0 5 10 15 20 25 30 35 40 45 50 55 MB
I-01
II-02
I-03
II-04
I-05
II-06
I-07
II-08
I-09
II-10
I-11
II-12
I-13
II-14
I-15
II-16
I-17
II-18
I-19
II-20
I-21
II-22
I-23
II-24
I-25
II-26
I-27
II-28
I-29
II-30
I-31
SYN
INV
TRANS
DUP
INV+DUP
INV+TRANS
ALLSPE

Supplementary Fig. 18. Comparisons between intersubgenome homoeologous chromosomes. Only regions larger than 5 kb are shown. SYN: synteny region; TRANS: translocation region; INV: inversion region; INVTR: inversion and translocation region; DUP: duplication region, including duplication loss and duplication gain; INVDP: inversion and duplication region; ALLSPE: allele-specific region. The heatmap tracks for each chromosome represent the density of tandem repeats (non-overlap window size = 10 kb) identified by Tandem Repeat Finder, which indicate the location of centromeric regions. The histogram tracks for each chromosome represent the density of enrichment 13-mer sequence (non-overlap window size = 100 kb). Orange for subgenome A, cyan for subgenome B. Source data are provided as a Source Data file.

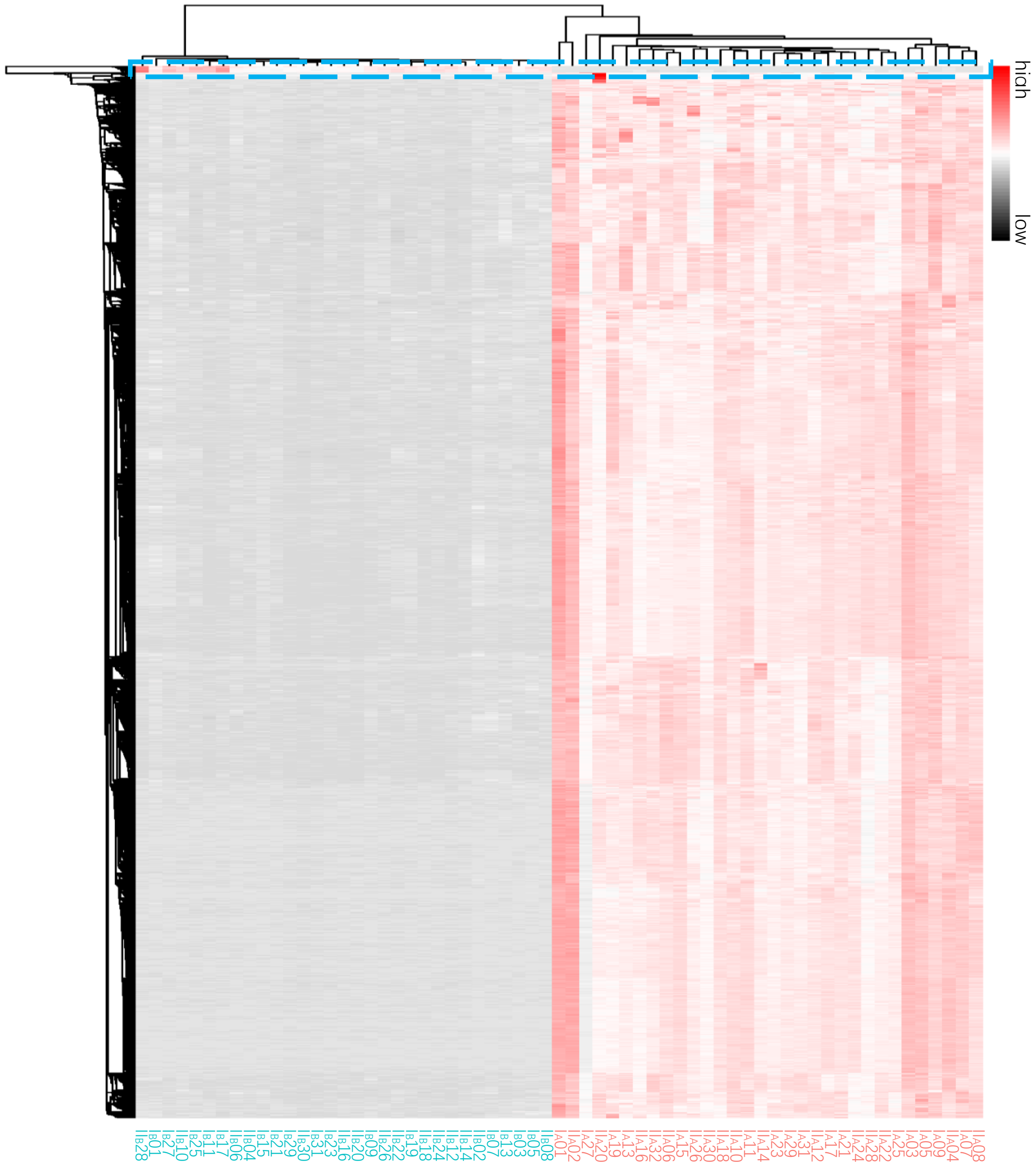

Supplementary Fig. 19. Clustering of all enriched 13-mers for their counts in each chromosome.

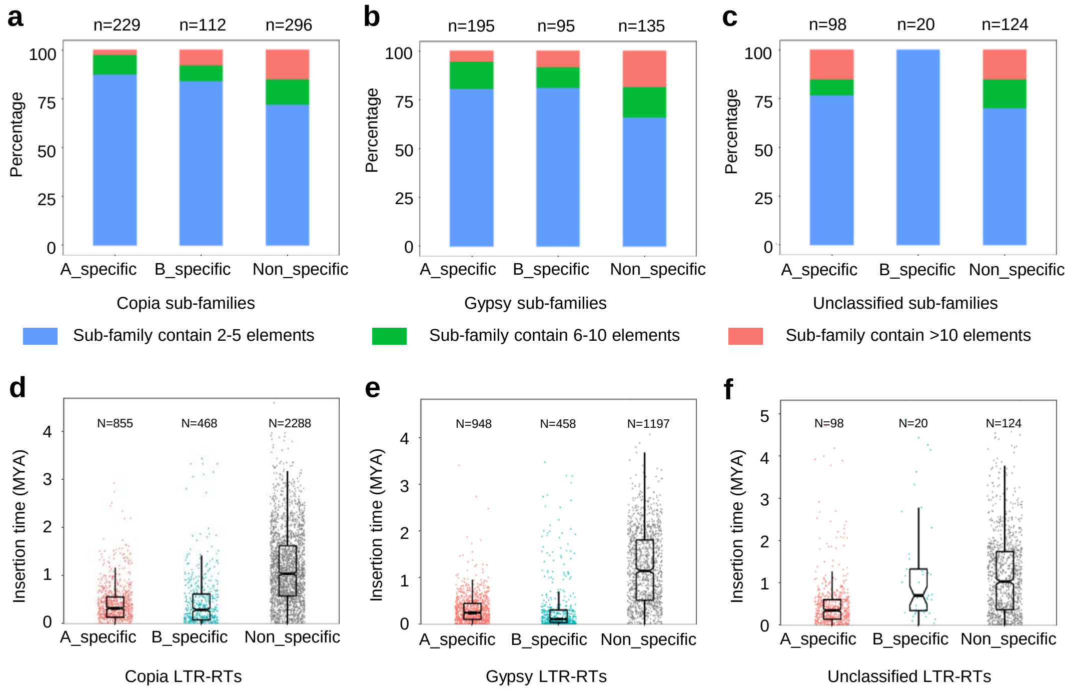

Supplementary Fig. 20. Comparisons between LRT-RT sub-families belonging to A subgenome specific, B subgenome specific and non specific groups. a-c, The percentages of sub-families containing 2 to 5 elements, 6 to 10 elements and more than 10 elements were compared between A subgenome specific, B subgenome specific and non specific groups for Copia (a), Gypsy (b) and Unclassified (c) LTR-RTs. **d-f,** Insertion times of elements belonging to A subgenome specific, B subgenome specific and non specific sub-families are shown for Copia (d), Gypsy (e) and Unclassified (f) LTR-RTs. The boxplot shows the median, 25-75% range and 1.5× interquartile range.

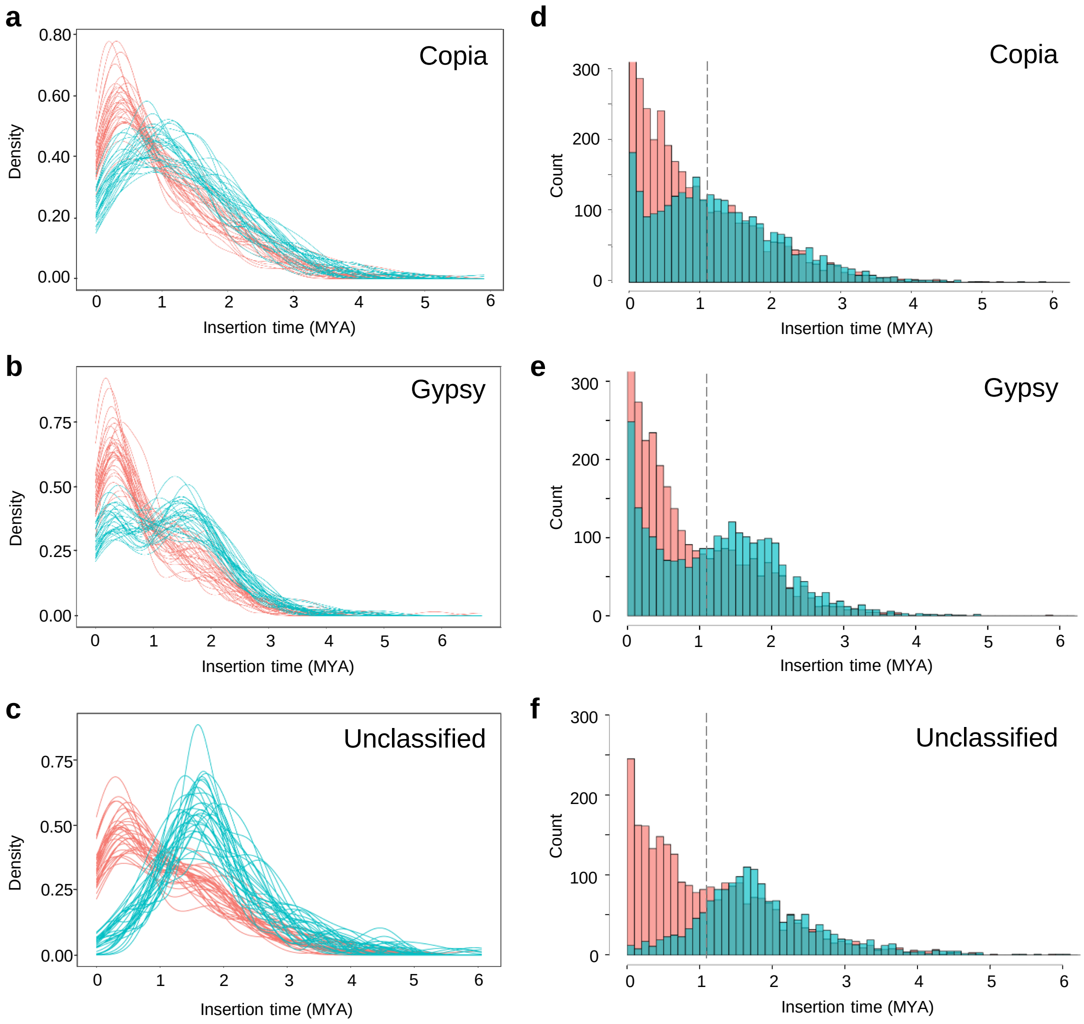

Supplementary Fig. 21. Insertion time distribution of different LTR-RT superfamilies. a-d, Kernel density plots for the insertion time of Copia (a), Gypsy (b), Unclassified (c) LTR-RTs. **d-f,** Count histograms for the insertion time of Copia (d), Gypsy (e), Unclassified (f) LTR-RTs.

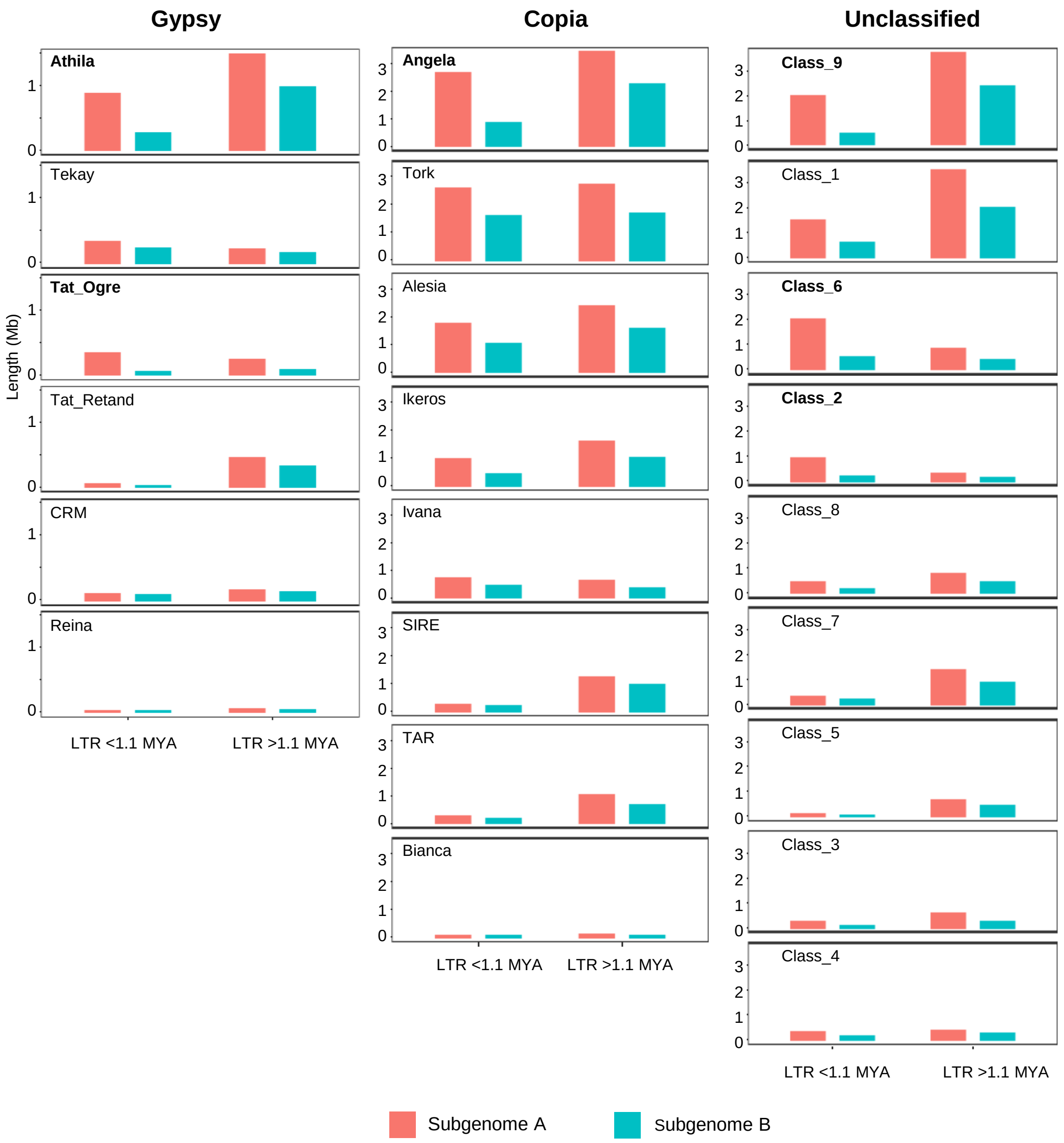

Supplementary Fig. 22. Comparisons of the length of LTR-RT lineages inserted before and after 1.1 MYA between the subgenomes A and B.

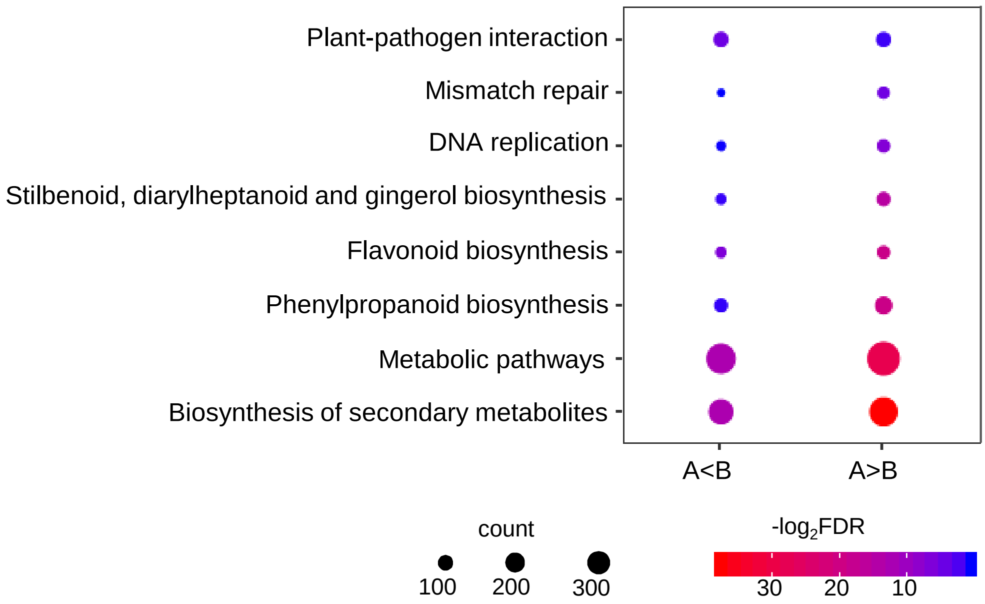

Supplementary Fig. 23. Enriched pathways for genes from inter-chromosome syntenic gene pairs that were significantly highly expressed in subgenome A or subgenome B.

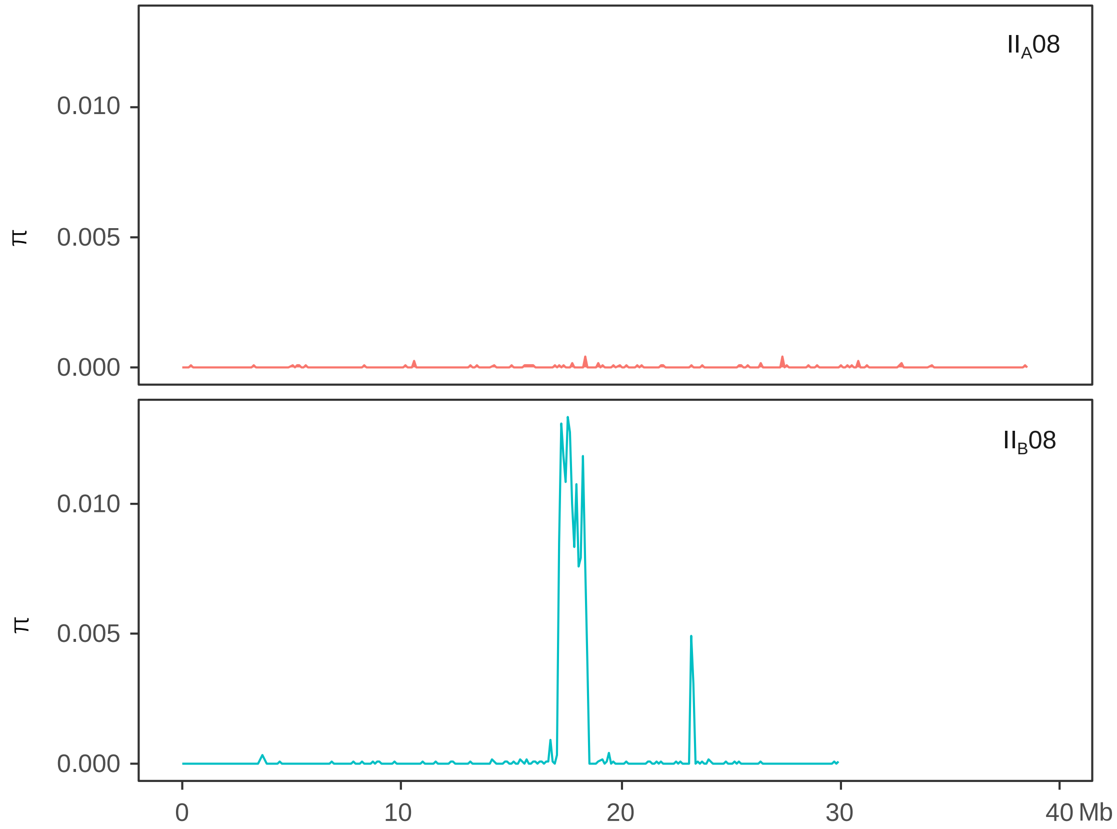

Supplementary Fig. 24. Distribution of genetic diversity (π value) along chromosomes II_A08 and II_B08. The π values in continuous 100 kb windows are shown. Source data are provided as a Source Data file.

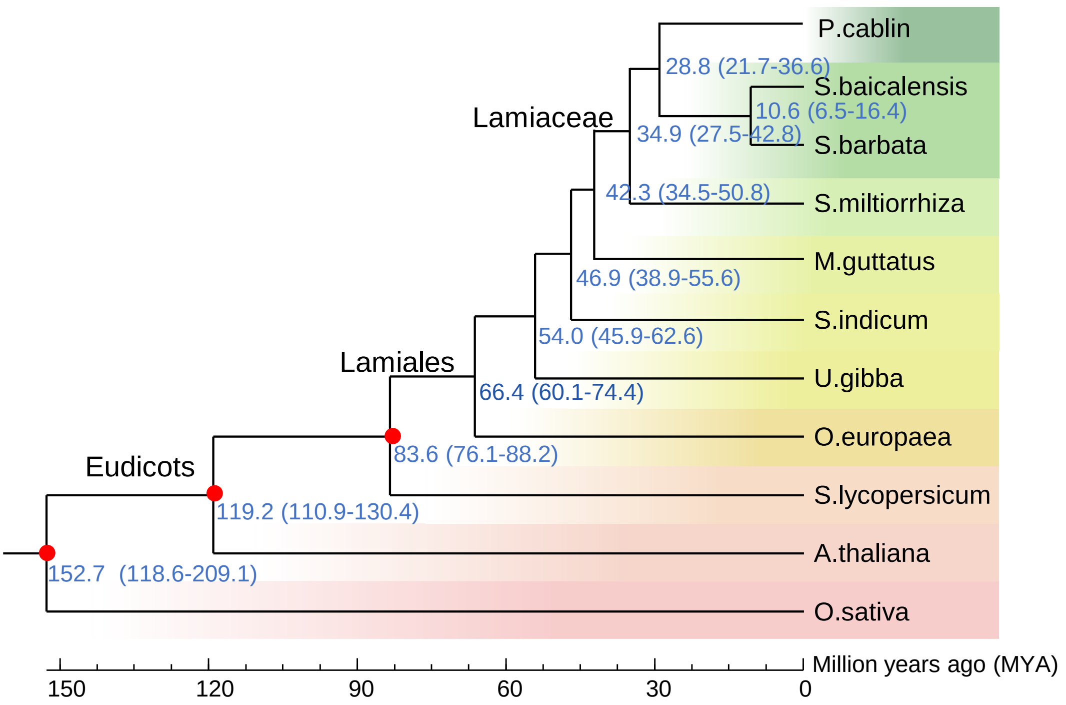

Supplementary Fig. 25. Phylogenetic analysis and divergence time estimations based on 11 plant species. Branch lengths are proportional to divergence times. Divergence times (MYA) were indicated by mean and 95% highest posterior density intervals beside the branch nodes. The red node indicated the calibrations in divergence time estimation. All nodes in the tree are fully supported by the ML bootstrap results (BS=100%).

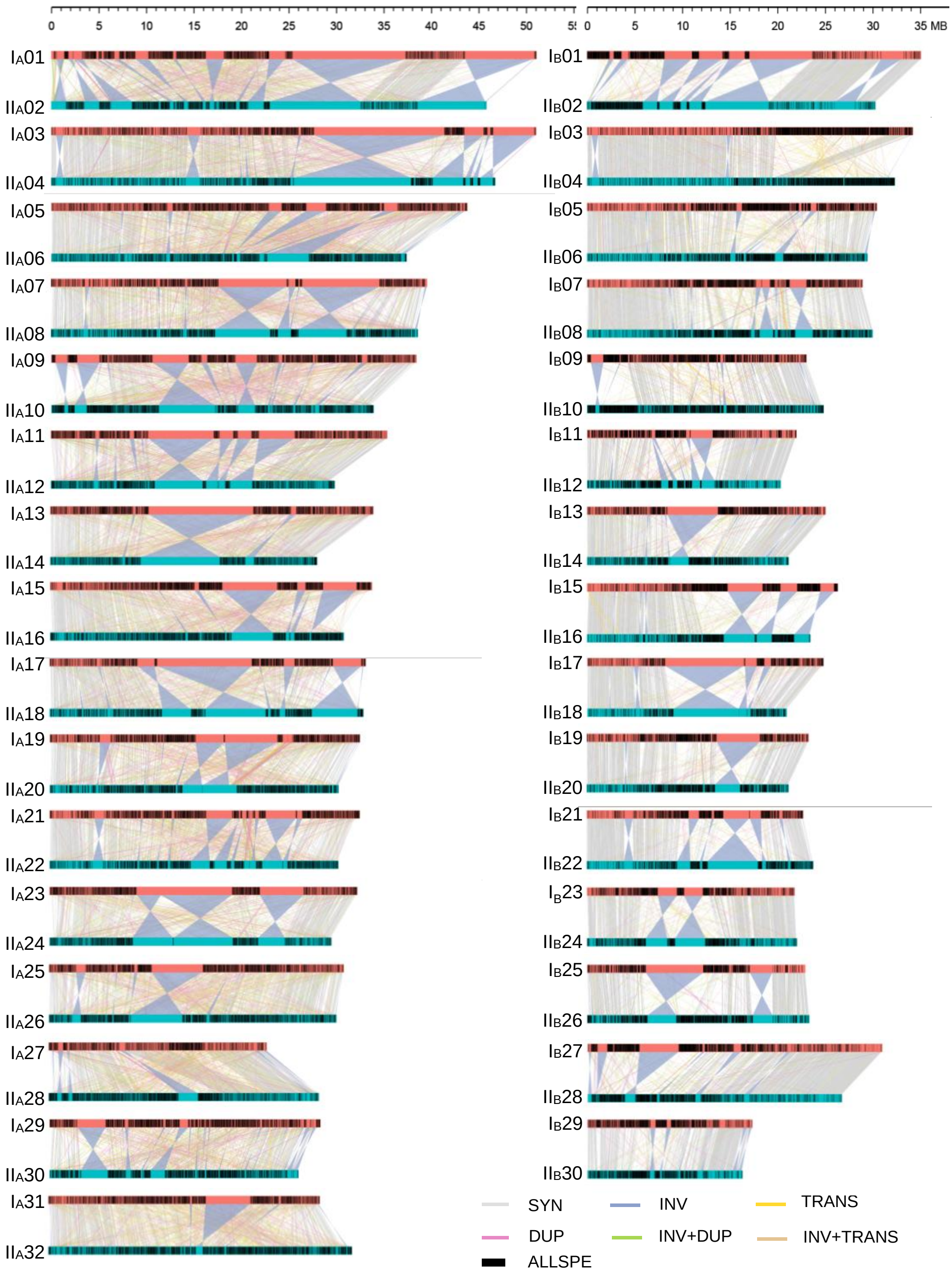

Supplementary Fig. 26. Comparisons between intrasubgenome homoeologous chromosomes. Only regions larger than 5 kb are shown. SYN: synteny region; TRANS: translocation region; INV: inversion region; INVTR: inversion and translocation region; DUP: duplication region, including duplication loss and duplication gain; INVDP: inversion and duplication region; ALLSPE: allele-specific region. Source data are provided as a Source Data file.

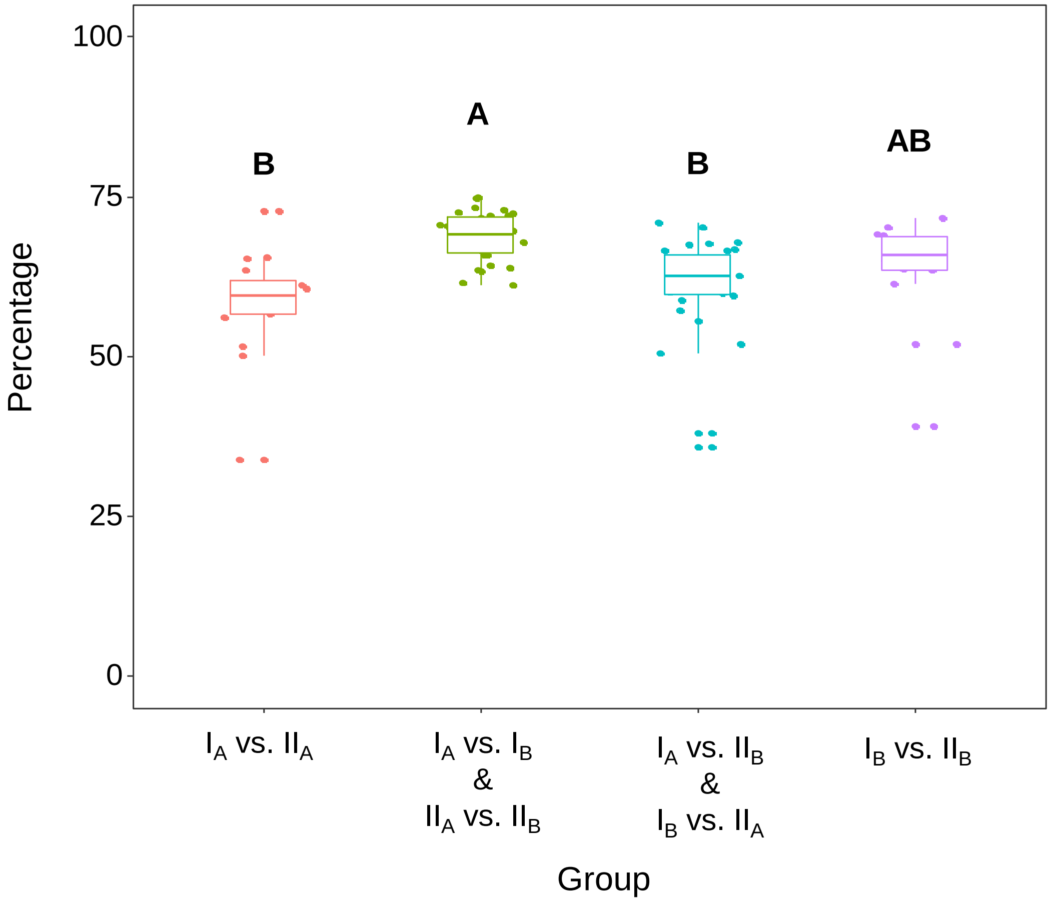

Supplementary Fig. 27. Comparisons of the percentage of syntenic genes between homoeologous chromosomes from any two mini-subgenomes. Different letters at the top of each column indicate significant differences by ANOVA (Tukey HSD test, $p < 0.001$). The boxplot shows the median and 25-75% range and 1.5× interquartile range. Percentage of syntenic genes from N=16, N=31, N=31 and N=15 homoeologous chromosome pairs were examined respectively for four groups from left to right. Source data are provided as a Source Data file.

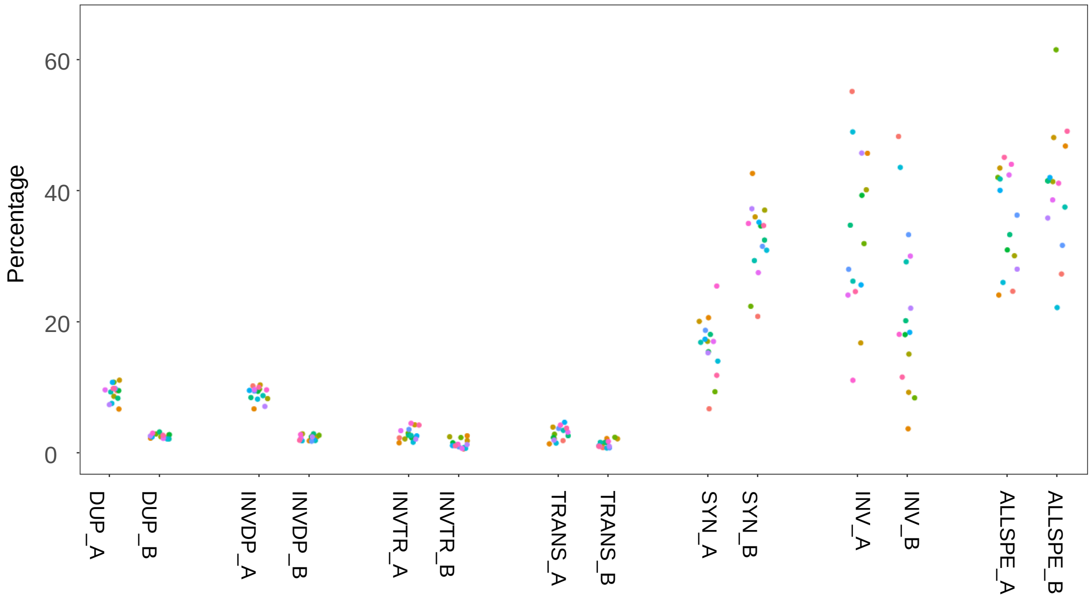

Supplementary Fig. 28. Comparisons of the length percentage of different structure variation types between intrasubgenome homoeologous chromosomes. SYN: synteny region; TRANS: translocation region; INV: inversion region; INVTR: inversion and translocation region; DUP: duplication region, including duplication loss and duplication gain; INVDP: inversion and duplication region; ALLSPE: allele-specific region.

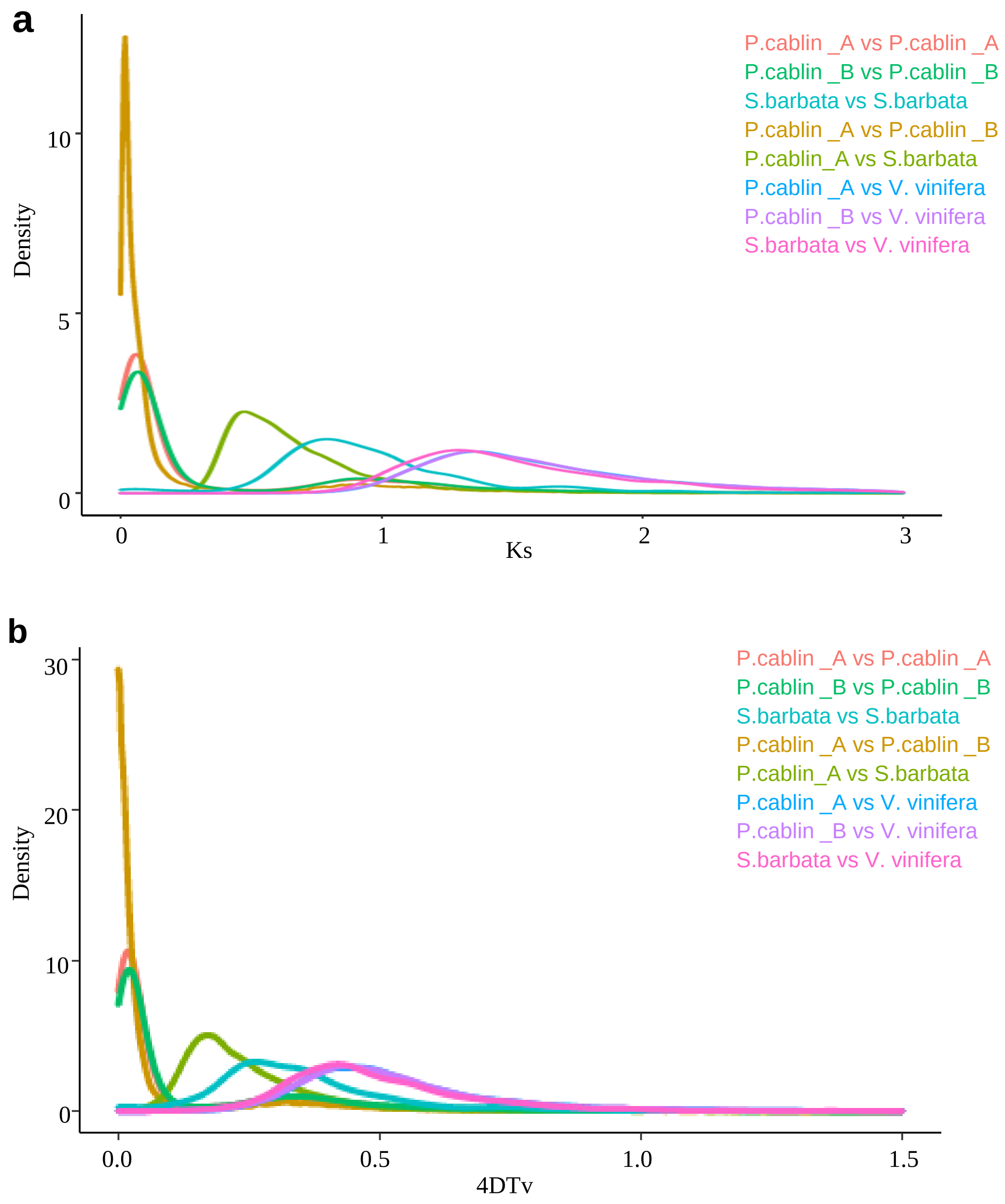

Supplementary Fig. 29. Kernel density plots for original Ks (a) and 4DTv (b) between genomes among patchouli subgenome A, patchouli subgenome B, *Scutellaria barbata* and *Vitis vinifera* genomes.

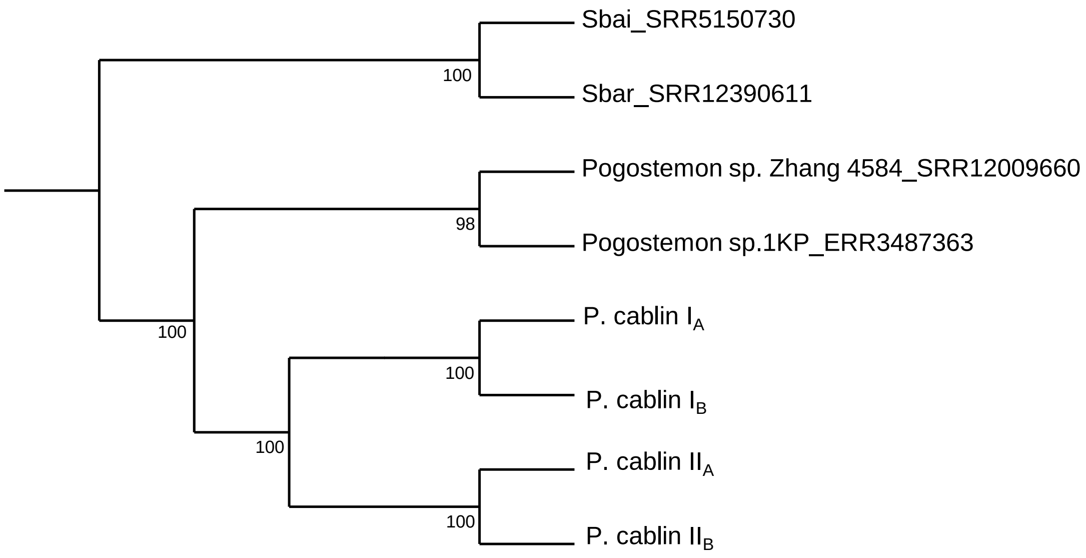

Supplementary Fig. 30. Maximum likelihood tree constructing by transcriptome data for other congeners in *Pogostemon*. Support values are from 100 bootstrap replicates.

Supplementary Table 1. Statistics for sequence data produced in this project.

Sequence type	Target	Data description					
NovaSeq	Survey+ Assembly	Insert size (bp)	Total base (bp)	Read length (bp)	Q20 (%)	Q30 (%)	GC (%)
		≤400	58,803,472,200	150;150	97.50;92.68	92.54;83.76	34.64;35.06
PacBio	Assembly	Total base (Gb)	Subreads base (Gb)	Subreads number	Subreads N50	Subreads mean length	P1 ratio
		166.68	166.6	8,141,755	31,512	20,462	81.16%
Hi-C	Anchor	Test reads pair	Test unique Di-tags	Valid interaction ratio	Total reads	Read length (bp)	Total base (bp)
		3,896,613	1,037,621	26.63%	903,333,182	150;150	135,499,977,300
Nanopore	Validation	Q>7 base (Gb)	Q>7 reads number	Q>7 reads N50 (bp)	Q>7 reads mean length (bp)	>100 kb reads base (bp)	>100 kb reads number
		23.34	908,139	52,945	25,696	4,727,771,972	35,101
Re-seq	Genetic diversity	Sample name	Insert size (bp)	Clean base (bp)	Read length (bp)	Q30 (%)	GC (%)
		YC	≤400	42,737,899,800	150;150	93.40;90.34	36.15;36.26
		HN	≤400	46,849,105,200	150;150	94.42;93.15	36.85;36.84
		YN	≤400	40,836,955,200	150;150	93.64;92.16	37.21;37.28
		SP	≤400	41,015,835,600	150;150	92.44;87.89	36.28;36.68
		GY	≤400	41,364,758,300	150;150	94.20;90.25	36.05;36.09

Supplementary Table 2. Genome size and gene number statistics for species of Lamiales.

Species	Genome size (bp)	Gene number	Transcript number	Mean mRNA length (bp)	Mean CDS length (bp)	Mean exon per transcript
Salvia miltiorrhiza	537,834,812	30,478	30,478	1,418	1,173	5.4
Scutellaria baicalensis	378,940,171	28,953	28,953	1,439	1,126	5.2
Scutellaria barbata	337,649,164	24,338	28,739	1,629	1,296	6.1
Milulus guttatus	322,166,964	29,525	31,861	1,880	1,352	6.3
Sesamum indicum	275,059,498	26,075	35,323	2,140	1,412	7.5
Utricularia gibba	100,688,548	29,666	29,666	900	900	6.2
Olea europaea	24,392,457	44,493	58,334	2,096	1,259	7.1

Supplementary Table 3. Statistics for genes of each TPS family.

Type	Duplicated_gene	Total_gene	Percentage
TPS-a	100	144	69.44%
TPS-a-I	34	41	82.93%
TPS-a-II	30	47	63.83%
TPS-a-III	36	56	64.29%
TPS-b	52	79	65.82%
TPS-c	4	12	33.33%
TPS-e/f	8	17	47.06%
TPS-g	8	14	57.14%
TOTAL	172	266	64.66%

Supplementary Table 4. Protein sequence similarity between seven tandemly duplicated and one reported *PatPTS* genes.

PatPTS	100							
Pat_ 49G071800	99.64	100						
Pat_ 49G072000	99.82	99.82	100					
Pat_ 49G072100	100	99.64	99.82	100				
Pat_ 49G072200	100	99.64	99.82	100	100			
Pat_ 49G072400	100	99.64	99.82	100	100	100		
Pat_ 49G072500	100	99.64	99.82	100	100	100	100	
Pat_ 49G072600	97.96	97.74	97.96	97.96	97.96	97.96	97.96	100
	PatPTS	Pat_ 49G071800	Pat_ 49G072000	Pat_ 49G072100	Pat_ 49G072200	Pat_ 49G072400	Pat_ 49G072500	Pat_ 49G072600

Supplementary Table 5. Content of patchouli alcohol in three patchouli accessions.

Accession	Sample	Peak_area	Content (mg/g FW)
SP	SP-1	666,851	0.244
SP	SP-2	680,891	0.246
SP	SP-3	580,985	0.216
YN	YN-1	7,341,029	2.836
YN	YN-2	9,440,747	2.927
YN	YN-3	7,833,034	2.811
HN	HN-1	12,656,445	4.747
HN	HN-2	10,591,166	4.338
HN	HN-3	14,315,784	5.921

Supplementary Table 6. Statistics for all repeat sequences in the patchouli genome.

Repeat type				bp Masked*	% Masked
TE	Class I: Retrotransposon	LTR-RT	Gypsy	463,247,414	23.89%
			Copia	235,483,382	12.14%
			Others	167,074,045	8.61%
		Non-LTR	LINE	27,076,154	1.39%
	Class II: DNA transposon	Subclass I (TIR)	Mutator	17,318,933	0.89%
			hAT	6,677,179	0.34%
			CACTA	3,043,506	0.16%
			PIF-Harbinger	3,136,661	0.16%
			Tcl-Mariner	705,392	0.03%
			Others	164,539,725	8.48%
		Subclass II	Helitron	7,953,772	0.41%
	Unknown			181,278,624	9.35%
Tandem Repeat	Low_complexity			11,543	0.00%
	Satellite			104,543	0.01%
	Simple_repeat			1,475,057	0.08%

* Full-length and truncated fragments of repeats were both included.

Supplementary Table 7. LTR assembly index (LAI) values of chromosomes in larger and smaller subgenomes.

Larger subgenome	LAI	Smaller subgenome	LAI
Chr01	19.478	Chr11	5.121
Chr04	19.288	Chr29	5.687
Chr02	18.406	Chr12	6.172
Chr03	19.005	Chr20	5.268
Chr05	19.158	Chr26	5.744
Chr09	19.717	Chr34	7.713
Chr06	20.243	Chr35	5.533
Chr07	18.901	Chr32	5.943
Chr08	19.460	Chr50	4.968
Chr14	18.633	Chr44	5.235
Chr10	19.928	Chr55	5.286
Chr31	22.277	Chr60	4.709
Chr13	18.818	Chr43	5.436
Chr39	20.322	Chr58	5.785
Chr15	18.348	Chr41	5.798
Chr25	18.128	Chr47	5.010
Chr16	19.977	Chr45	6.028
Chr17	22.144	Chr59	5.323
Chr18	18.175	Chr49	6.510
Chr27	18.591	Chr57	6.260
Chr19	17.713	Chr53	6.346
Chr28	18.147	Chr46	5.166
Chr21	15.845	Chr56	5.952
Chr33	19.343	Chr54	5.419
Chr23	20.372	Chr52	5.154
Chr30	18.876	Chr48	5.039
Chr51	21.923	Chr24	5.098
Chr38	18.469	Chr40	5.505
Chr37	19.390	Chr62	5.735
Chr42	18.438	Chr63	6.054
Chr36	20.967	Chr61	6.314
Chr22	19.553		

Supplementary Table 8. Statistics for structural variations between intersubgenome homoeologous chromosomes.

Variation type	Subgenome A length (bp)	Subgenome B length (bp)	Total length (bp)	Percentage of whole genome
SYN	501,021,262	443,961,512	944,982,774	49.50%
DUP	41,448,311	32,616,885	74,065,196	3.88%
INVDP	36,536,828	27,521,825	64,058,653	3.36%
TRANS	22,816,313	19,844,688	42,661,001	2.23%
INV	222,612,672	155,649,603	378,262,275	19.81%
INVTR	16,789,342	14,949,094	31,738,436	1.66%
ALLSPE	262,874,350	110,425,238	373,299,588	19.55%

SYN: syntenic region;

TRANS: translocation region;

INV: inversion region;

INVTR: inversion and translocation region;

DUP: duplication region, contain duplication loss and duplication gain.

INVDP: inversion and duplication region;

ALLSPE: allele specific region;

Supplementary Table 9. Enrichment numbers for 13-mers in the permutation test of each intersubgenome homoeologous chromosomes.

Switch chromosome	Enrichment 13-mers count	
	Subgenome A	Subgenome B
I_A01-I_B01	0	0
II_A02-II_B02	0	0
I_A03-I_B03	0	0
II_A04-II_B04	0	0
I_A05-I_B05	0	0
II_A06-II_B06	1	0
I_A07-I_B07	0	0
II_A08-II_B08	1	0
I_A09-I_B09	0	0
II_A10-II_B10	0	0
I_A11-I_B11	0	0
II_A12-II_B12	1	0
I_A13-I_B13	0	0
II_A14-II_B14	3	0
I_A15-I_B15	0	0
II_A16-II_B16	0	0
IA17-IB17	5	0
II_A18-II_B18	0	0
I_A19-I_B19	0	0
II_A20-II_B20	0	0
I_A21-I_B21	0	0
II_A22-II_B22	0	0
I_A23-I_B23	0	0
II_A24-II_B24	0	0
I_A25-I_B25	1	0
II_A26-II_B26	1	0
I_A27-I_B27	5	0
II_A28-II_B28	1	0
I_A29-I_B29	0	0
II_A30-II_B30	0	0
I_A31-I_B31	2	0

Supplementary Table 10. Composition comparisons between intersubgenome homoeologous chromosomes.

Chr	Subgenome A length (bp)			Subgenome B length (bp)			Absolute length difference (bp)		
	Repeat	Gene	Other	Repeat	Gene	Other	Repeat	Gene	Other
I-01	33,578,666	8,283,369	9,108,880	20,516,471	7,407,125	7,181,429	13,062,195	876,244	1,927,451
II-02	29,268,853	8,345,587	8,105,053	16,841,530	7,242,625	6,221,179	12,427,323	1,102,962	1,883,874
I-03	30,767,099	11,028,282	9,169,350	17,068,388	9,740,949	7,425,833	13,698,711	1,287,333	1,743,517
II-04	26,170,242	10,993,606	9,499,354	14,328,222	9,728,534	8,275,745	11,842,020	1,265,072	1,223,609
I-05	27,848,191	8,838,247	7,017,206	16,842,493	7,515,356	6,082,647	11,005,698	1,322,891	934,559
II-06	22,343,676	8,442,010	6,534,093	14,957,407	7,795,174	6,691,857	7,386,269	646,836	157,764
I-07	25,323,927	7,158,945	7,052,359	16,202,041	6,539,798	6,142,754	9,121,886	619,147	909,605
II-08	24,396,603	7,460,001	6,705,057	17,018,440	6,761,813	6,163,902	7,378,163	698,188	541,155
I-09	26,387,408	5,693,028	6,276,681	13,467,076	4,623,723	4,900,750	12,920,332	1,069,305	1,375,931
II-10	22,710,601	5,396,373	5,755,991	14,802,474	5,022,562	4,956,867	7,908,127	373,811	799,124
I-11	23,644,378	6,108,055	5,781,819	12,703,298	4,879,938	4,340,677	10,941,080	1,228,117	1,441,142
II-12	18,836,263	5,609,907	5,543,169	11,363,669	4,669,531	4,231,509	7,472,594	940,376	1,311,660
I-13	22,005,942	6,322,048	5,638,122	14,757,453	5,489,787	4,764,431	7,248,489	832,261	873,691
II-14	17,757,123	5,500,389	4,780,076	11,611,106	4,979,383	4,544,992	6,146,017	521,006	235,084
I-15	20,618,983	7,074,469	6,112,707	13,785,465	6,393,551	6,141,934	6,833,518	680,918	29,227
II-16	18,493,267	6,499,372	5,848,165	12,240,533	5,864,624	5,318,254	6,252,734	634,748	529,911
I-17	20,478,500	6,551,003	6,153,345	14,224,642	5,634,789	4,920,591	6,253,858	916,214	1,232,754
II-18	20,977,241	6,343,357	5,617,498	11,092,186	5,222,579	4,592,627	9,885,055	1,120,778	1,024,871
I-19	21,794,446	5,502,541	5,284,332	13,460,088	5,146,367	4,629,206	8,334,358	356,174	655,126
II-20	19,617,473	5,613,258	5,094,814	11,910,435	4,861,108	4,365,307	7,707,038	752,150	729,507
I-21	21,173,186	5,551,375	5,849,426	12,671,707	5,133,676	4,888,771	8,501,479	417,699	960,655
II-22	19,540,403	5,720,616	5,056,785	13,717,046	5,320,848	4,711,111	5,823,357	399,768	345,674
I-23	20,960,390	5,909,375	5,401,944	11,937,802	5,129,341	4,654,256	9,022,588	780,034	747,688
II-24	18,942,804	5,669,165	4,944,852	12,254,773	4,992,872	4,721,836	6,688,031	676,293	223,016
I-25	20,306,533	5,695,901	4,951,085	13,250,744	5,117,848	4,437,798	7,055,789	578,053	513,287
II-26	19,779,499	5,430,574	4,965,256	13,924,458	4,962,075	4,354,181	5,855,041	468,499	611,075
I-27	12,128,081	6,004,901	4,735,003	18,254,166	6,307,495	6,318,457	6,126,085	302,594	1,583,454
II-28	17,031,276	6,242,747	5,092,559	15,316,204	6,077,763	5,248,544	1,715,072	164,984	155,985
I-29	19,090,841	4,680,803	4,661,633	9,573,228	4,089,997	3,570,542	9,517,613	590,806	1,091,091
II-30	16,645,726	4,834,740	4,635,306	8,741,079	3,929,846	3,535,305	7,904,647	904,894	1,100,001
I-31	18,013,479	5,403,921	5,039,002	10,707,814	4,551,722	4,417,978	7,305,665	852,199	621,024
Total							259,340,832	23,380,354	27,512,512

Supplementary Table 11. Total element length (bp) of DNA transposon and different LTR-RT families in subgenomes A and B.

	LTR_Copia	LTR_Gypsy	LTR_unclassify	DNA_transposon
subgenome A	134,328,968	274,600,056	94,291,669	109,749,198
Subgenome B	81,599,384	155,957,392	61,810,134	86,881,647

* The II_A32 were excluded from subgenome A for equally comparison.

Supplementary Table 12. Comparisons of the counts of LTR-RT lineages inserted before and after 1.1 MYA between subgenomes A and B.

Type		<1.1 MYA		>1.1 MYA		*P*-value*
		Subgenome A	Subgenome B	Subgenome A	Subgenome B	
Gypsy	Athila	752	112	387	525	< 2.2E-16
	CRM	72	90	48	83	1.90E-01
	Reina	158	56	87	98	4.25E-08
	Tat_Ogre	182	12	51	66	< 2.2E-16
	Tat_Retand	35	13	114	186	7.16E-06
	Tekay	660	801	201	275	2.65E-01
Copia	Alesia	556	313	275	263	2.08E-06
	Angela	134	36	86	129	8.78E-15
	Bianca	17	6	11	14	0.04507
	Ikeros	328	60	159	182	< 2.2E-16
	Ivana	285	100	90	91	2.42E-08
	SIRE	15	14	64	99	2.25E-01
	TAR	104	19	126	120	1.87E-10
	Tork	669	720	237	340	0.004607
Unclassified	Class_1	265	50	226	321	< 2.2E-16
	Class_2	254	5	33	36	< 2.2E-16
	Class_3	16	7	25	27	0.1307
	Class_4	122	40	56	57	1.98E-05
	Class_5	19	4	58	62	0.002713
	Class_6	219	13	74	81	< 2.2E-16
	Class_7	44	11	113	115	3.66E-05
	Class_8	215	7	66	90	< 2.2E-16
	Class_9	85	23	138	177	2.00E-10

*: Fisher's exact test were used for comparisons using 95 percent confidence interval and two-sided alternative hypothesis. No multiple comparison was performed.

Supplementary Table 13. Statistics for structural variations between intrasubgenome homoeologous chromosomes.

Variation type	I chromosome length (bp)	II chromosome length (bp)	Total length (bp)	Percentage in whole genome
SYN	211,453,151	211,161,857	422,615,008	21.48%
DUP	60,625,542	60,195,587	120,821,129	6.14%
INVDP	58,967,439	57,934,570	116,902,009	5.94%
TRANS	21,197,273	21,244,475	42,441,748	2.16%
INV	287,380,799	242,369,352	529,750,151	26.93%
INVTR	21,011,370	20,943,209	41,954,579	2.13%
ALLSPE	353,558,257	339,001,036	692,559,293	35.21%

SYN: synteny region;

TRANS: translocation region;

INV: inversion region;

INVTR: inversion and translocation region;

DUP: duplication region, contain duplication loss and duplication gain.

INVDP: inversion and duplication region;

ALLSPE: allele specific region;

Supplementary Table 14. Reads remapping summary of whole genome sequence data for other congeners in *Pogostemon.*

Species	SRA accession	Original reads no.	Mapped reads no.	Map percentage	Genome coverage
Pogostemon brevicorollus	SRR7121980	443,805,189	45,444,398	10.24%	6.43%
Pogostemon nigrescens	SRR7121772	481,274,844	92,482,947	19.22%	6.94%

Supplementary Table 15. Centromeric repeat sequences.

Repeat	Sequence
Pat_cen1	AAGCTTGCGCCATAGAGTCACTAAAAACTCAAAGTGCAGAGCTGATTCTACTCCGTG<br>AGCCAGGTCAAGAACTCCTTGAAGTGAGCATTTTCTTGCCATTTTTATGGTCAAATTA<br>GTTATTATACATGAGAATATAGTTGGAGAACATGTTTTCAGAGTATAAAAATGCTAGG
Pat_cen2	CAAAATGAAGGAAATATGCATCGAAACGAAGAAAATATTGATCGTAACGAGAAAATG<br>AGCATCAAAATGAAGGAAATATGCATCAAAAAGAAAAAAATATTGATCGTAACAAGA<br>AAATGATCATCAAAATGAAGGAAATATGCATCAAAACGAAGAAAATATTGATCGTAA<br>ATA
