# Supplementary material for: Chromosome-level and haplotype-resolved genome provides insight into the tetraploid hybrid origin of patchouli
Source: Nat Commun. 2022 Jun 18;13:3511. doi: 10.1038/s41467-022-31121-w (PMC9206139; doi:10.1038/s41467-022-31121-w)
Supplement: Supplementary file 2 — Description of Additional Supplementary Files [file 41467_2022_31121_MOESM2_ESM.pdf]

Description of Additional Supplementary Files

File Name: Supplementary Data 1
Description: SNP statistic for whole genome sequence of patchouli accesssion from Indonesia

File Name: Supplementary Data 2
Description: Heatmap of read number percentage belonging to each chromosome in a SLAF-seq dataset containing 22 patchouli accessions and in NovaSeq reads produced by this project

File Name: Supplementary Data 3
Description: Chromosome ID corresponding to before and after genome haplotying
