# Supplementary material for: Chromosome-level and haplotype-resolved genome provides insight into the tetraploid hybrid origin of patchouli
Source: Nat Commun. 2022 Jun 18;13:3511. doi: 10.1038/s41467-022-31121-w (PMC9206139; doi:10.1038/s41467-022-31121-w)
Supplement: Supplementary file 6 — Reporting Summary [file 41467_2022_31121_MOESM6_ESM.pdf]

# nature portfolio

Corresponding author(s): Yanting Shen

Last updated by author(s): May 28, 2022



# Reporting Summary

Nature Portfolio wishes to improve the reproducibility of the work that we publish. This form provides structure for consistency and transparency in reporting. For further information on Nature Portfolio policies, see our Editorial Policies and the Editorial Policy Checklist.

## Statistics

For all statistical analyses, confirm that the following items are present in the figure legend, table legend, main text, or Methods section.

| n/a | Confirmed | |
|---|---|---|
| ☐ | ☒ | The exact sample size ($n$) for each experimental group/condition, given as a discrete number and unit of measurement |
| ☐ | ☒ | A statement on whether measurements were taken from distinct samples or whether the same sample was measured repeatedly |
| ☐ | ☒ | The statistical test(s) used AND whether they are one- or two-sided<br>*Only common tests should be described solely by name; describe more complex techniques in the Methods section.* |
| ☒ | ☐ | A description of all covariates tested |
| ☐ | ☒ | A description of any assumptions or corrections, such as tests of normality and adjustment for multiple comparisons |
| ☐ | ☒ | A full description of the statistical parameters including central tendency (e.g. means) or other basic estimates (e.g. regression coefficient) AND variation (e.g. standard deviation) or associated estimates of uncertainty (e.g. confidence intervals) |
| ☐ | ☒ | For null hypothesis testing, the test statistic (e.g. $F$, $t$, $r$) with confidence intervals, effect sizes, degrees of freedom and $P$ value noted<br>*Give P values as exact values whenever suitable.* |
| ☒ | ☐ | For Bayesian analysis, information on the choice of priors and Markov chain Monte Carlo settings |
| ☒ | ☐ | For hierarchical and complex designs, identification of the appropriate level for tests and full reporting of outcomes |
| ☒ | ☐ | Estimates of effect sizes (e.g. Cohen's $d$, Pearson's $r$), indicating how they were calculated |

*Our web collection on statistics for biologists contains articles on many of the points above.*

## Software and code

Policy information about availability of computer code

| | |
|---|---|
| Data collection | 1. A mature Pogostemon cablin (Blanco) Benth. branch was collected from medicinal botanical garden (Yaowang Moutain) at Guangzhou University of Chinese Medicine and then tissue cultured for propagation. Young leaves from seedlings were used for genome sequencing. High-molecular-weight DNA was extracted and then sequenced on the PacBio Sequel II (SMRT) and Nanopore PromethION (ONT) platforms. The former was performed at Berry Genomic Corporation, Ltd (Beijing, China) by constructing a 40-kb SMRTbell library and the latter was performed at GrandOmics Biosciences (Wuhan, China) by constructing a 1D library. For Hi-C sequencing, DNA was manipulated after leaves were fixed with 1% (vol/vol) formaldehyde, cell lysis, chromatin digestion (DpnII), proximity-ligation treatments and DNA recovery as previously described. For NovaSeq, DNA was directly isolated using the Plant Genomic DNA Kit (DP305, Tiangen, Beijing, China) according to the manufacturer's protocol. Both Hi-C library and NovaSeq DNA PCR-free library were constructed and sequenced on an Illumina NovaSeq6000 system.<br>2. Cutting seedlings from patchouli plants originally collected from YangChun (YC), HaiNan (HN), ShiPai (SP), GaoYao (GY) and Indonesia (YN) were planted in our greenhouse at Guangzhou University of Chinese Medicine (Guangzhou, China). Their DNA was also extracted and sequenced on an Illumina NovaSeq6000 system. |
| Data analysis | Jellyfish (v2.3.0)<br>GenomeScope2.0<br>Minimap2 (version 2.5-r572)<br>Canu (v2.0)<br>smrtlink (v8.0)<br>Pilon (v1.22)<br>3D-DNA (v180419)<br>Juicebox (v1.11.08)<br>Mummer (ver 3.0) |

BWA (v0.7.16a-r1181)
seqkit (v0.13.0)
LTR_retriever (v2.8.7)
RepeatMasker (v4.1.0)
RepeatModeler (v2.0)
Fastp (version 0.20.1)
HISAT2 (v2.1.0)
Stringtie (v2.1.3b)
PASA (v2.4.1)
Genoma (v1.6.1)
SNAP (version 2006-07-28)
GeneMark-ESSuite (version 4.57)
Augustus (v3.2.2)
EVidenceModeler (v1.1.1)
InterProScan (v5.18-57.0)
CpGAVAS2
RNAmmer (v1.2)
tRNAscan-SE (v2.0.0)
Infernal (v1.1.2)
MUMmer (ver 3.0)
MCScanX
Blastp (v2.6.0)
SYRI (v1.4)
OrthoMCL (v2.0.9)
MAFFT (v7.471)
RAxML (v8.0.19)
synonymous_calc.py (https://github.com/tanghaibao/bio-pipeline/tree/master/synonymous_calculation)
TimeTree (http://timetree.org/)
Tephra (version 0.13.1)
Pfam database (ftp://ftp.ebi.ac.uk/pub/databases/Pfam)
FastTree (v2)
Viridiplantae_v3.0
BWA (v0.7.16a-r1181)
GATK (version 4.1.8.1)
VCFtools (v0.1.16)
HISAT2
StringTie (v2.1.3b)
KOBAS 3.0
MegaX (version 10.0.5)
MUSCLE (v3.8.31)
TandemRepeatFinder (v4.09)
ASTRAL-III
Phyparts (v0.0.1)
MITE Hunter (11-2011)
FlowJo7.6

For manuscripts utilizing custom algorithms or software that are central to the research but not yet described in published literature, software must be made available to editors and reviewers. We strongly encourage code deposition in a community repository (e.g. GitHub). See the Nature Portfolio guidelines for submitting code & software for further information.

# Data

Policy information about availability of data

All manuscripts must include a data availability statement. This statement should provide the following information, where applicable:

- Accession codes, unique identifiers, or web links for publicly available datasets
- A description of any restrictions on data availability
- For clinical datasets or third party data, please ensure that the statement adheres to our policy

1.All the sequencing data used in the genome assembly have been deposited into the Genome Sequence Archive (GSA) database in BIG Data Center under Accession Number CRA004172. Information for the assembled genome Patchouli_v1.0 was deposited both into the Genome Warehouse (GWH) (GWHBAZF00000000) database in the BIG Data Center and Genome database in the National Center for Biotechnology Information (NCBI) under the accession JAHCLQ000000000 (Submission ID: SUB7789864). Source data are provided as a Source Data file.
2. Eighteen Patchouli RNA-seq datasets (SRR8769986, SRR7268115 , SRR7268117, SRR8785265, SRR1770488, SRR7268119, SRR8756845, SRR7345998, SRR7345999, SRR7346000, SRR8755904, SRR8767850, SRR8755475, SRR8775235, SRR8775238, SRR8793583, SRR8809556, SRR8820010) were downloaded from the NCBI SRA database.
3.The sequences of Oryza sativa (GCF_001433935.1_IRGSP-1.0), Olea europaea (742605.1_O_europaea_v1), Sesamum indicum (GCF_000512975.1_S_indicum_v1.0) and Milulus guttatus (GCF_000504015.1_Mimgu1_0) were downloaded from the NCBI database, and those of Scutellaria barbata (GWHAOTP00000000) and Scutellaria baicalensis (GWHAOTC00000000) were downloaded from the Genome Warehouse, that of Salvia miltiorrhiza was downloaded from ftp://danshen.ndctcm.org:10402 that of Arabidopsis thaliana TAIR10), Solanum lycopersicum (ITAG 3.2) were downloaded from Phytozome, that of Utricularia gibba (ID29027) was downloaded from CoGe.

4. SLAF-seq reads for 22 patchouli accessions were downloaded from the NCBI SRA database (SRP057143)

# Field-specific reporting

Please select the one below that is the best fit for your research. If you are not sure, read the appropriate sections before making your selection.

- [x] Life sciences
- [ ] Behavioural & social sciences
- [ ] Ecological, evolutionary & environmental sciences

For a reference copy of the document with all sections, see nature.com/documents/nr-reporting-summary-flat.pdf

# Life sciences study design

All studies must disclose on these points even when the disclosure is negative.

| | |
|---|---|
| Sample size | 1. We sequence and de-novo assembled one patchouli genome.<br>2. We re-sequenced five patchouli accessions.<br>3. SLAF-seq reads for 22 patchouli accessions downloaded from the NCBI SRA database were used to confirm the representativeness of our patchouli genome.<br>4. 18 patchouli RNA-seq datasets downloaded from the NCBI SRA database were used for gene annotation and gene expression analysis.<br>5. Three patchouli accessions were used in GC-MS and RT-PCR experiments to measure patchouli alcohol content and PatTPS expression.<br>These sample sizes was sufficient for their related studies. |
| Data exclusions | There were no data excluded in our analysis. |
| Replication | 1. Each sample was repeated three times independently in flow cytometry experiment to measure genome size. All the result from the repeat experiments were similar.<br>2. The chromosome tableting experiments for chromosome counting were repeated more than 5 times. All the result from the repeat experiments were same.<br>3. The maximum-likelihood tree for for 11 species was constructed by RAxML and for TPS was constructed by MegaX when bootstrap was set to 1000. The bootstraps were all 100% as state in figure legend of Supplementary Fig. 25<br>4. GC-MS to measure patchouli alcohol content in three patchouli accessions were repeated three times. All the result from the repeat experiments were similar.<br>5. RT-PCR to measure expression of PatTPS in three patchouli accessions were repeated three times. All the result from the repeat experiments were similar. |
| Randomization | 1. To test the sensitivity of k-mer enrichment method in genome phasing, we performed permutation tests by exchanging the corresponding subgenome for chromosomes in one syntenic pair and then detected the enriched 13-mers again. |
| Blinding | Blind experiment is not required for our work. |

# Reporting for specific materials, systems and methods

We require information from authors about some types of materials, experimental systems and methods used in many studies. Here, indicate whether each material, system or method listed is relevant to your study. If you are not sure if a list item applies to your research, read the appropriate section before selecting a response.

## Materials & experimental systems

| n/a | Involved in the study | |
|---|---|---|
| x | | Antibodies |
| x | | Eukaryotic cell lines |
| x | | Palaeontology and archaeology |
| x | | Animals and other organisms |
| x | | Human research participants |
| x | | Clinical data |
| x | | Dual use research of concern |

## Methods

| n/a | Involved in the study | |
|---|---|---|
| x | | ChIP-seq |
| | x | Flow cytometry |
| x | | MRI-based neuroimaging |

# Flow Cytometry

## Plots

Confirm that:

☒ The axis labels state the marker and fluorochrome used (e.g. CD4-FITC).

☒ The axis scales are clearly visible. Include numbers along axes only for bottom left plot of group (a 'group' is an analysis of identical markers).

☒ All plots are contour plots with outliers or pseudocolor plots.

☒ A numerical value for number of cells or percentage (with statistics) is provided.

## Methodology

| | |
|---|---|
| Sample preparation | Young leaves of sequenced patchouli plants and a tomato plant used for a control were chopped with a sharp razor blade in OTTO I buffer (45 mM $MgCl_2 \cdot 6H_2O$, 20 mM MOPS, 30 mM sodium citrate, 1% (W/V) PVP-40, 0.2% (v/v) Tritonx-100, 10 mM $Na_2EDTA$, 20 µL/mL β-mercaptoethanol; pH 7.5). Samples were incubated for 5 min and filtered through 70 um Biosharp filters. Plant cell nuclei were stained by adding 1.5 mL of OTTO II buffer containing 500 uL of propidium iodide and RNAse A in the dark for 20-30 min. |
| Instrument | BD FACSAria III flow cytometer |
| Software | FlowJo7.6 |
| Cell population abundance | We generally collect a total of 5000-10000 cells, and measure the purity of the target cell group and the integrity of the nucleus by the CV (coefficient of variation) value in the histogram. Usually the purity of the target cell group and the integrity of the nucleus were considered reliable when CV<5%. |
| Gating strategy | No gating was applied. The analysis of the plant genome size results in a non-gated one parameter histogram output on a linear scale. |

☐ Tick this box to confirm that a figure exemplifying the gating strategy is provided in the Supplementary Information.
