# Supplementary material for: Chromosome-level and haplotype-resolved genome provides insight into the tetraploid hybrid origin of patchouli
Source: Nat Commun. 2022 Jun 18;13:3511. doi: 10.1038/s41467-022-31121-w (PMC9206139; doi:10.1038/s41467-022-31121-w)
Supplement: Supplementary file 7 — Source Data [file 41467_2022_31121_MOESM7_ESM.zip › Source Data 2.docx]

The uncropped scans of SDS-PAGE gel in Supplementary Fig. 14a. The first five lanes were displayed in Supplementary Fig. 14a.
